# Leveraging shared ancestral variation to detect local introgression

**DOI:** 10.1371/journal.pgen.1010155

**Published:** 2024-01-08

**Authors:** Lesly Lopez Fang, David Peede, Diego Ortega-Del Vecchyo, Emily Jane McTavish, Emilia Huerta-Sánchez

**Affiliations:** 1 Department of Life & Environmental Sciences, University of California, Merced, Merced, California, United States of America; 2 Quantitative & Systems Biology Graduate Group, University of California, Merced, Merced, California, United States of America; 3 Department of Ecology, Evolution and Organismal Biology, Brown University, Providence, Rhode Island, United States of America; 4 Center for Computational Biology, Brown University, Providence, Rhode Island, United States of America; 5 Institute at Brown for Environment and Society, Brown University, Providence, Rhode Island, United States of America; 6 Laboratorio Internacional de Investigación sobre el Genoma Humano, Universidad Nacional Autónoma de México, Santiago de Querétaro, Querétaro, México; Case Western Reserve University, UNITED STATES

## Abstract

Introgression is a common evolutionary phenomenon that results in shared genetic material across non-sister taxa. Existing statistical methods such as Patterson’s *D* statistic can detect introgression by measuring an excess of shared derived alleles between populations. The *D* statistic is effective to detect genome-wide patterns of introgression but can give spurious inferences of introgression when applied to local regions. We propose a new statistic, *D*^+^, that leverages both shared ancestral and derived alleles to infer local introgressed regions. Incorporating both shared derived and ancestral alleles increases the number of informative sites per region, improving our ability to identify local introgression. We use a coalescent framework to derive the expected value of this statistic as a function of different demographic parameters under an instantaneous admixture model and use coalescent simulations to compute the power and precision of *D*^+^. While the power of *D* and *D*^+^ is comparable, *D*^+^ has better precision than *D*. We apply *D*^+^ to empirical data from the 1000 Genome Project and *Heliconius* butterflies to infer local targets of introgression in humans and in butterflies.

## Author summary

Characterizing how pervasive introgression is across the tree of life is an outstanding question in evolutionary biology. To address this question, we need to detect and quantify introgression to investigate how natural selection has acted on introgressed genetic variation. The *D* statistic is a widely used method to detect introgression at the genome level, but this method cannot accurately detect introgression locally in the genome. To improve its performance at the local level, we incorporate ancestral variation shared between the donor and recipient populations. We show, theoretically and with simulations, that re-introduced ancestral alleles into the recipient population also contain information to detect introgression. Using both shared derived and ancestral variation, we define a new statistic, *D*^+^, that can be used to detect the location of introgressed regions in a genome with a history of introgression.

## Introduction

Analyses of both modern and ancient DNA have revealed that introgression is a common evolutionary process in the history of many species. Introgression has been found in swordtail fish [1], *Heliconius* butterflies [2,3], and from Neanderthals and Denisovans to modern-day non-African populations [4–8] as well as many other systems. These observations suggest that introgression is pervasive and thus determining its relative contribution to the evolution of a species is of evolutionary interest [9]. Therefore, detecting and quantifying introgressed segments in the genome is necessary to begin measuring its biological importance. Introgression may introduce both adaptive and deleterious variation in the recipient population. For example, Tibetans inherited a beneficial haplotype at the *EPAS1* gene from Denisovans through gene flow that facilitated high altitude adaptation to the hypoxic environment in the Tibetan plateau [10–13] which is an example of adaptive introgression—positive selection acting on introgressed variants [10,14–16]. Similarly, purifying selection has also acted on introgressed variation [17–20] to remove deleterious introgressed variants and under specific conditions can mimic signatures of adaptive introgression [18,21].

**Fig 1:**
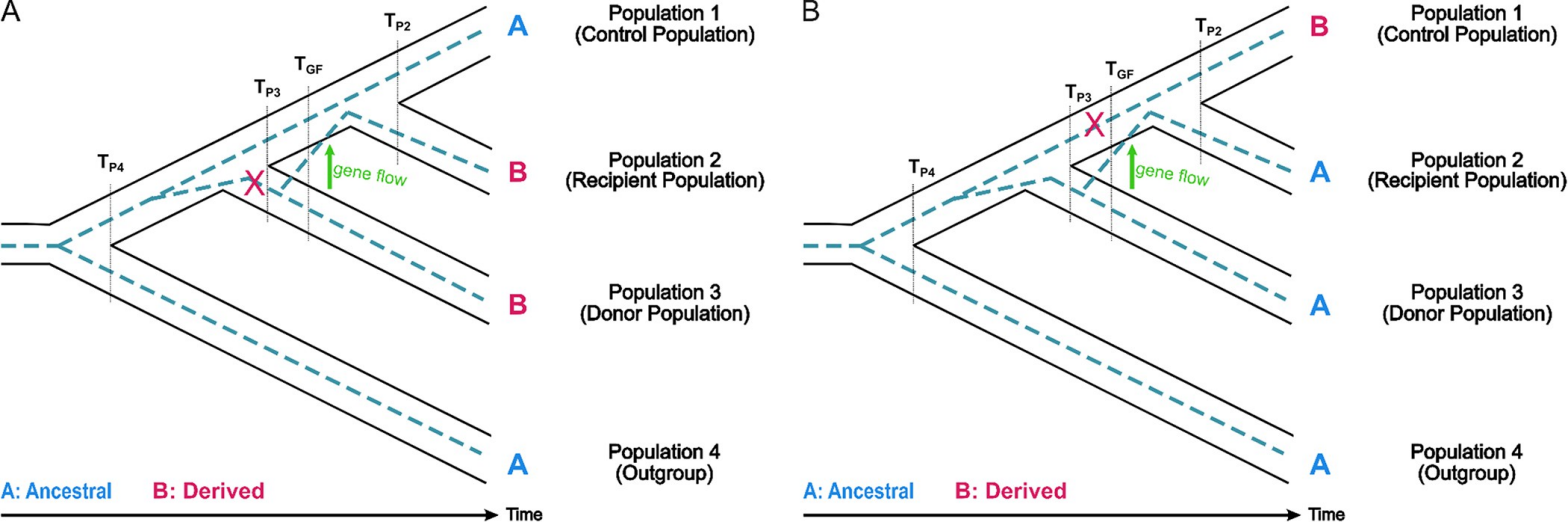
Species and gene trees depicting informative sites due to gene flow. (A) Shared derived allele between population 2 and population 3, or ABBA site, and (B) shared ancestral allele between population 2 and population 3, or BAAA site, due to gene flow from population 3 to population 2. The ancestral allele is denoted A and the derived allele is denoted B. T_P4_ is the time of divergence between population 4 and the ancestral population of population 1, population 2 and population 3. T_P3_ is the time of divergence between population 1 and the ancestral population of population 1 and population 2. T_P2_ is the time of divergence between population 1 and population 2. T_GF_ denotes the time of gene flow from donor population to recipient population.

The most widely-used method to detect introgression using data from one or more individuals from each of four populations is the ABBA-BABA statistic, also known as Patterson’s *D* statistic [4,5]. This statistic has been used to detect introgression from Neanderthals and Denisovans into modern humans [4,22,23] as well as other systems. The *D* statistic uses species tree and gene tree discordances within a 4-population tree with two potential targets of introgression defined as population 1 (P_1_) and population 2 (P_2_); a donor population (P_3_) as the source of gene flow to P_1_ or P_2_, and an outgroup population (P_4_, see Fig 1A and 1B). The patterns of biallelic single nucleotide polymorphisms (SNP) generated by these gene trees (dotted lines in Fig 1A and 1B) provide information on the shared ancestry between lineages in each population. The *D*-statistic looks at patterns when the gene tree does not match the species/population tree, which can be due to chance through Incomplete Lineage Sorting (ILS) or gene flow from the donor population into P_1_ or P_2_. While ILS will generate an equal number of discordant sites shared between P_3_ and P_1_ and P_3_ and P_2_, introgression will result in an excess of shared sites between P_3_ and either P_1_ or P_2_. *D* is a measure of this excess number of shared derived alleles.

The *D* statistic was designed to detect genome-wide gene flow but has also been used to look for signals of gene flow in local regions of the genome. However, studies have found that *D* produces spurious inferences of gene flow when applied to areas of the genome with low nucleotide diversity [24,25]. A previous study [25] partitioned butterfly genomes into small 5 kb windows and computed the *D* statistic in each window which showed that the *D* statistic becomes more unreliable when considering windows of low nucleotide diversity, because the variance of *D* is maximized in these windows. To improve inference of introgression in small windows [25] propose a new statistic, ${\hat{f}}_{d}$, that is a better estimator of the true introgression proportion. More recently [24] proposed to improve the *D* statistic by including the number of sites with an BBAA pattern—which is reduced in the presence of introgression—in the denominator of the *D* statistic.

In this study, we propose a new statistic, *D*^+^, to detect introgression in genomic windows. In addition to using the shared derived variation measured in the *D* statistic, *D*^+^ also leverages shared ancestral variation between the donor population and the recipient population. Introgression introduces not only mutations that accrued in the donor population before the gene flow event, but also re-introduces ancestral alleles in the recipient population. Following [5], we derive the theoretical expectations for the *D*^+^ statistic under a coalescent framework to study its properties as a function of the admixture proportion. We use simulations to measure its power, false positive rate and precision compared to the *D* statistic. We also measure its performance by applying it to humans and butterflies. We find that the *D*^+^ statistic is more precise at detecting introgressed regions than the *D* statistic due to its lower false positive rate in small genomic regions, making it a useful statistic to identify local targets of introgression.

## Methods

### *D*^+^ statistic

Patterson’s *D* statistic uses species and gene tree discordance within a 4-population tree with two populations as potential targets of introgression, population 1 (P_1_) and population 2 (P_2_). Population 3 (P_3_) is a source of gene flow to either P_1_ or P_2_, and population 4 (P_4_) serves as an outgroup (Fig 1). The patterns of biallelic single nucleotide polymorphisms (SNP) generated by the gene trees provide information on the shared ancestry between lineages in each population. Both the *D* and *D*^+^ statistic look at site patterns yielded when the gene tree does not match the species tree. A mutation will convert an ancestral allele (A), determined by the allele present in the outgroup, into a derived allele (B). An ABBA site (Fig 1A) describes a derived allele shared between P_3_ and P_2_, while a BABA site occurs when a derived allele is shared between P_3_ and P_1_. An ABBA or BABA site could arise due to incomplete lineage sorting (ILS) or gene flow. Under coalescent expectations, incomplete lineage sorting will generate equal numbers of gene trees with ABBA or BABA sites. An ABBA site can only be generated in a gene tree where P_3_ and P_2_ coalesce first before they find a common ancestor with P_1_. On the other hand, a BABA site only occurs on gene trees where P_1_ and P_3_ coalesce first before they find a common ancestor with P_2_. We expect an excess of ABBA sites when there is gene flow from P_3_ to P_2_.

The *D* statistic measures an excess of ABBA or BABA sites [4,5]. *D* is the normalized difference between ABBA and BABA sites, $D=\frac{\sum_{i=1}^{L}{ABBA}_{i}-{BABA}_{i}}{\sum_{i=1}^{L}{ABBA}_{i}+{BABA}_{i}}$. The *D* statistic assumes that the frequency of ABBA and BABA sites due to ILS is approximately equal. Therefore, an excess of shared derived sites between P_3_ and P_2_, or ABBA sites, indicates gene flow from P_3_ to P_2_ as shown in Fig 1A. Conversely, an excess of BABA sites indicates gene flow from P_3_ to P_1_.

We extend this idea by making use of the fact that introgressed regions are inherited in chunks that contain both shared derived alleles and ancestral alleles that are introduced into the recipient population. *D*^+^ leverages the shared ancestral alleles between P_3_ to P_2_ to increase the amount of data about shared genetic variation in low nucleotide diversity regions. Sites where the ancestral allele is shared between P_3_ and P_2_ and the derived allele is only found in P_1_ are BAAA sites (Fig 1B). In ABAA sites the ancestral allele is shared between P_3_ and P_1_ while P_2_ has a derived allele. *D*^+^ incorporates both shared derived alleles and ancestral alleles to strengthen our inferences of introgression.


$$D^{+}=\frac{\sum_{i=1}^{L}\left({ABBA}_{i}-{BABA}_{i})+(BAAA_{i}-ABAA_{i}\right)}{\sum_{i=1}^{L}\left({ABBA}_{i}+{BABA}_{i})+(BAAA_{i}+ABAA_{i}\right)}$$ (1)

When the sample size is bigger than one, we can write down the equation for *D*^+^ as a function of the observed derived allele frequencies in populations P_1_, P_2_, P_3_ or P_4_. If the frequency of the allele at site i for population j is ${\hat{p}}_{ij}$, and we have L sites,

$$D^{+}=\frac{\sum_{i=1}^{L}\left(\left(1-{\hat{p}}_{i1}){\hat{p}}_{i2}{\hat{p}}_{i3}(1-{\hat{p}}_{i4})-{\hat{p}}_{i1}(1-{\hat{p}}_{i2}){\hat{p}}_{i3}(1-{\hat{p}}_{i4}\right))+({\hat{p}}_{i1}(1-{\hat{p}}_{i2})(1-{\hat{p}}_{i3})({1-\hat{p}}_{i4})-(1-{\hat{p}}_{i1}){\hat{p}}_{i2}(1-{\hat{p}}_{i3})({1-\hat{p}}_{i4})\right)}{\sum_{i=1}^{L}\left(\left(1-{\hat{p}}_{i1}){\hat{p}}_{i2}{\hat{p}}_{i3}(1-{\hat{p}}_{i4})+{\hat{p}}_{i1}(1-{\hat{p}}_{i2}){\hat{p}}_{i3}(1-{\hat{p}}_{i4}\right))+({\hat{p}}_{i1}(1-{\hat{p}}_{i2})(1-{\hat{p}}_{i3})({1-\hat{p}}_{i4})+(1-{\hat{p}}_{i1}){\hat{p}}_{i2}(1-{\hat{p}}_{i3})({1-\hat{p}}_{i4})\right)}$$ (2)

While in this paper, we mostly focus on comparisons between *D*^+^ and *D*, note that we could also define a statistic *D*_*ancestral*_ that measures the excess of shared ancestral alleles between P_3_ and P_2_ in a similar manner that the *D* statistic measures an excess of shared derived alleles between P_3_ and P_2_:

$$D_{ancestral}=\frac{\sum_{i=1}^{L}{BAAA}_{i}-{ABAA}_{i}}{\sum_{i=1}^{L}{BAAA}_{i}+{ABAA}_{i}}$$ (3)

*D*_*ancestral*_ is normalized and ranges from -1 to 1, with *D*_*ancestral*_ = 1 indicating gene flow from P_3_ to P_2_ and *D*_*ancestral*_ = −1 indicating gene flow from P_3_ to P_1_. *D*_*ancestral*_ approximates zero under the null hypothesis of no gene flow.

Durand et al. (2011) used a coalescent framework to derive the expectation of the *D* statistic under an instantaneous admixture model (IUA). The probability of getting an ABBA or BABA site is dependent on the mutation rate and the expected branch length of the branch where a mutation yields an ABBA site (T_ABBA_) or the branch where a mutation yields a BABA site (T_BABA_). The mutation rate μ is assumed to be constant. Therefore, the expected number of ABBA or BABA sites can be estimated by calculating the expectation of branch lengths of T_ABBA_ and T_BABA_ and multiplying by the mutation rate [5]. Similarly, we can compute the probability of getting an ABAA or BAAA site (see S1 Appendix), and we derived the expected lengths of T_BAAA_ and T_ABAA_ following the same framework. The full derivation of the expectation of T_BAAA_ and T_ABAA_ is in S1 Appendix. We find that the analytical expectation of *D*^+^ is

$$E[D^{+}]=\frac{\left(\mu *E[T_{ABBA}]-\mu *E[T_{BABA}])+(\mu *E[T_{BAAA}]-\mu *E[T_{ABAA}]\right)}{\left(\mu *E[T_{ABBA}]+\mu *E[T_{BABA}])+(\mu *E[T_{BAAA}]+\mu *E[T_{ABAA}]\right)}.$$ (4)

As is true of ABBA and BABA sites, the expected number of BAAA and ABAA sites are equal when there is no gene flow. This is because, under no gene flow, we expect a similar amount of ancestral allele sharing between P_1_ and P_3_ and between P_2_ and P_3_. In the case of the BAAA and ABAA sites, we expect a similar amount of BAAA and ABAA sites under no gene flow assuming the same mutation rate in P_1_ and P_2_. As the admixture proportion from P_3_ to P_2_ increases, the number of BAAA sites exceeds the number of ABAA sites. The expected difference is a function of the admixture proportion *f* and the branch lengths of T_P3_ and T_GF_:

$$E[T_{ABBA}-T_{BABA}]=E[T_{BAAA}-T_{ABAA}]=f(T_{P3}-T_{GF})$$ (5)

### Simulations to verify theoretical results for n = 1

**Fig 2:**
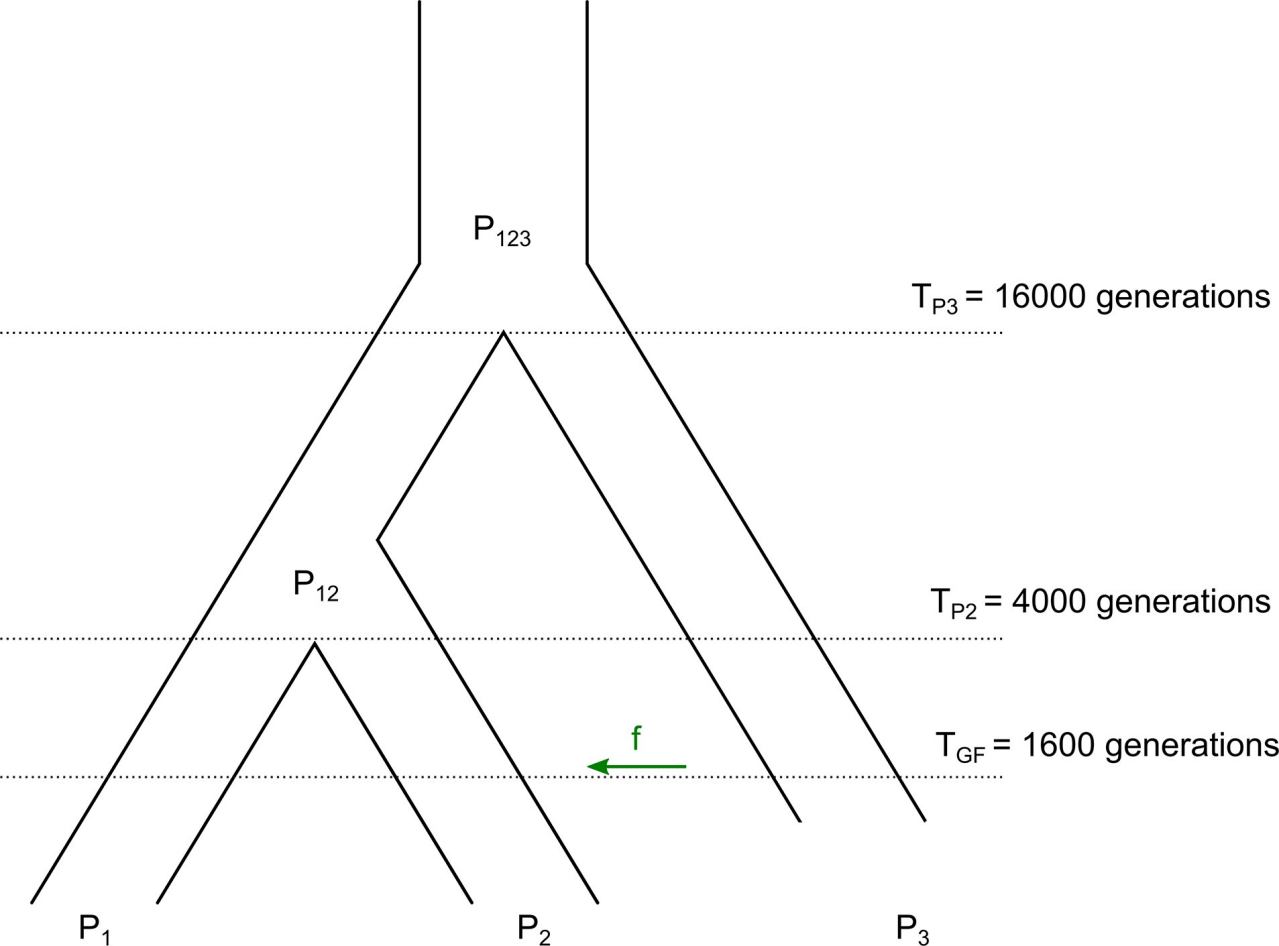
Demographic model for msprime simulations. (P_1_) and (P_2_) are sister populations that are closely related to (P_3_). P_1_ and P_2_ diverged at time T_P2_ (4,000 generations ago) and the ancestral population of P_1_ and P_2_ (P_12_) diverged from P_3_ at time T_P3_ (16,000 generations ago). There is gene flow from (P_3_) to (P_2_) at time T_GF_ (1,600 generations ago) with an admixture proportion *f* = 3%. Divergence time of populations shown follow the demography of modern humans.

**Fig 3:**
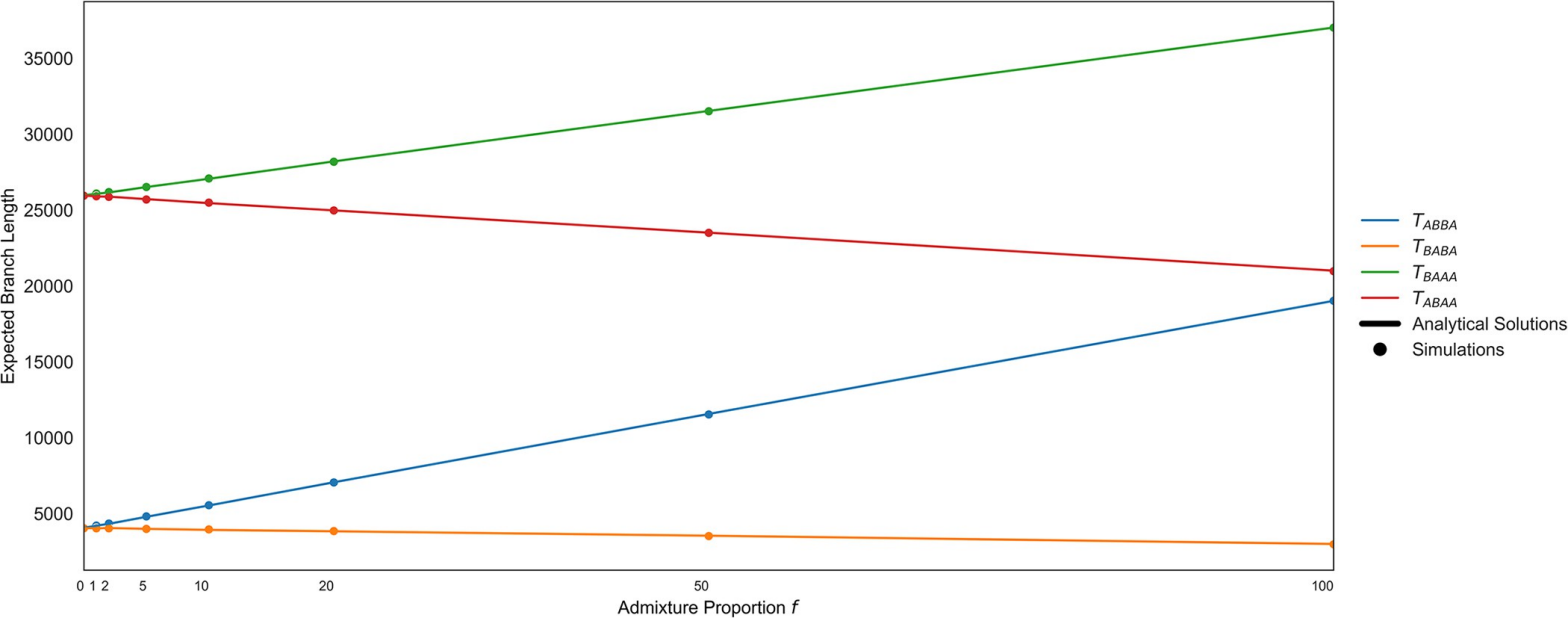
Analytical and simulated expected branch lengths of T_ABBA_, T_BABA_, T_BAAA_ and T_ABAA_. The analytical (lines) and simulated (dots) expected branch lengths of T_ABBA_, T_BABA_, T_BAAA_ and T_ABAA_ for different proportions of admixture *f* between P_3_ and P_2_. The solutions to the analytical expectations match the simulated expectations. The branch length of T_ABBA_ is the branch that would produce an ABBA site pattern. The expectation of T_ABBA_ (E[T_ABBA_]) can be used to calculate the expected number of ABBA sites. The same is true for T_BABA_, T_BAAA_, and T_ABAA_ for their respective site patterns. With no admixture (*f* = 0) the expected branch lengths for ABBA and BABA sites are equal (E[T_ABBA_] = E[T_BABA_]), as are the expected branch lengths for BAAA and ABAA sites (E[T_BAAA_] = E[T_ABAA_]) because the number of ABBA sites equals BABA sites and the number of BAAA sites equals the number ABAA sites due to ILS. As the admixture proportion increases, the expectation of T_ABBA_ and T_ABBA_ increases due to excess ABBA and BAAA sites. The difference in T_BAAA_ and T_ABAA_ (T_BAAA_—T_ABAA_) is equal to the difference in T_ABBA_ and T_BABA_ (T_ABBA_—T_BABA_).

To verify the theoretical results under the demographic model depicted in Fig 2, we calculate the expected branch lengths of T_ABBA_, T_BABA_, T_BAAA_ and T_ABAA_ and expectation of *D* and *D*^+^ using mspms simulations (see Figs 3 and S4) for a range of admixture proportions (*f*). We ran 1,000,000 simulations of independent loci and averaged the branch lengths of T_ABBA_, T_BABA_, T_BAAA_ and T_ABAA_ from the Newick tree file output of each locus. The branches T_ABBA_, T_BABA_, T_BAAA_ and T_ABAA_ are the branches where a mutation would yield an ABBA, BABA, BAAA or ABAA site, illustrated in S1 Fig. We simulated small, independent loci with 250 sites per loci and a mutation rate of 10^−8^ per bp per generation and no recombination. We also calculated *D* and *D*^+^ from the number of ABBA, BABA, BAAA and ABAA sites per locus and averaged *D* and *D*^+^ across all 1,000,000 loci. An example mspms simulation command for 1,000,000 independent loci with 1 sample taken from each of the 4 populations for an admixture proportion of 3% is:

mspms 4 1000000 -t 0.1 -I 4 1 1 1 1 -es 0.1 2 0.97 -ej 0.1 5 3 -ej 0.25 1 2 -ej 0.5 2 3 -ej 20 3 4 –T.

### Simulations to benchmark *D*^*+*^ using n = 1 for all populations

To evaluate the precision and recall of *D* and *D*^+^ we ran coalescent simulations using the software msprime [26]. The simulations followed the model depicting the evolutionary history of modern humans (Fig 2). The African and Eurasian populations are P_1_ and P_2_, respectively, and P_3_ is the Neanderthal population. The African-Eurasian and Neanderthal divergence time T_P3_ was set 16,000 generations ago and the Eurasian and African divergence time T_P2_ was set 4,000 generations ago [16]. The time of gene flow (T_GF_) between Neanderthals and Eurasians was set 1,600 generations ago [16]. We use an admixture proportion (*f*) of 3%. All simulations had a constant N_e_ of 10,000, a mutation rate of 1.5*10^−8^ per bp per generation and a recombination rate of 10^−8^ per bp per generation following [16]. We ran 100 simulations of 20 MB genomes with n = 1 for P_1_, P_2_ and P_3_, and, in each run, we sampled a single haplotype to compute *D*^+^ using Eq 1. *D*^+^ can also be applied to populations with a sample size greater than 1. We ran 100 msprime simulations with n = 200 genomes for P_1_ and P_2_ and n = 2 for P_3_ and computed *D*^+^ using derived allele frequencies (Eq 2). The full code for simulations can be found in a GitHub repository (https://github.com/LeslyLopezFang/Dplus).

To evaluate the performance of *D*^+^ under different values for the admixture proportion, recombination rate and mutation rate under this demography, we ran 100 msprime simulations with the new parameter value for a 20 MB genome for n = 1 for P_1_, P_2_ and P_3_ under a model with no admixture and a model with admixture. We considered the following cases: f = 2%, 5% and 10%, a mutation rate of half the default mutation rate of 1.5*10^−8^ per bp per generation, a mutation rate that was double the default mutation rate of 1.5*10^−8^ per bp per generation, a recombination rate that was half the default recombination rate of 10^−8^ per bp per generation, and a recombination rate that was double the default recombination rate of 10^−8^ per bp per generation.

### Calculating precision, recall and false positive rate in simulated human data

**Fig 4:**
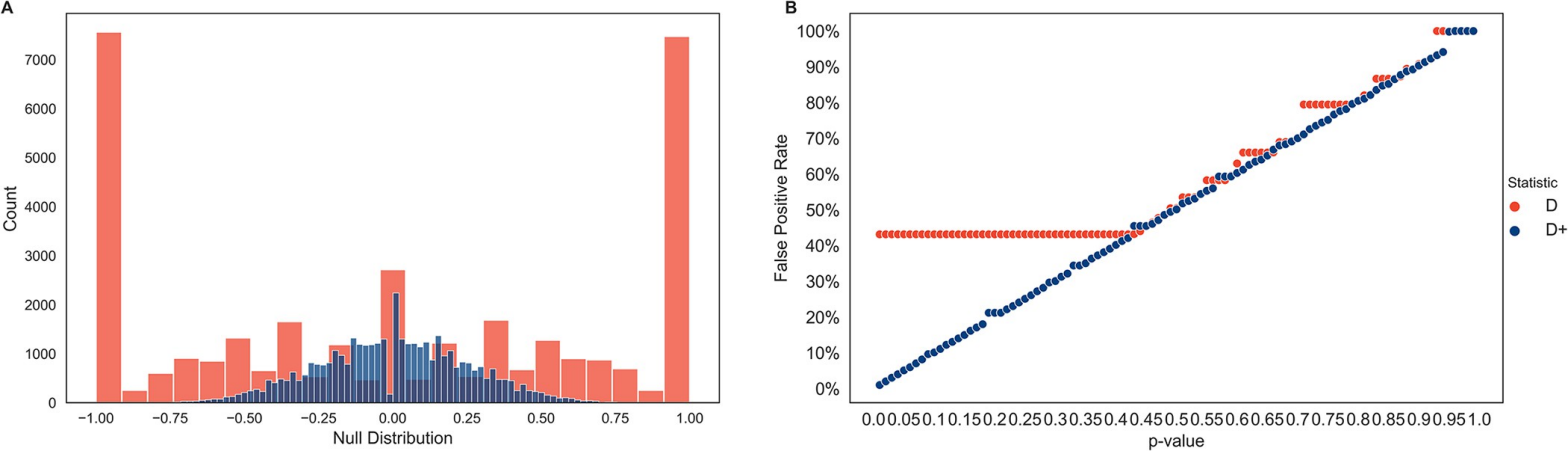
Null distribution and false positive rate for *D* and *D*^+^ in simulations with no gene flow. *D* and *D*^+^ were calculated in 50 kb windows of 100 runs of a 20 MB simulated genome under a model with no admixture. **(A)** The average of the null distribution of *D* and *D*^+^ is zero with a standard deviation of 0.74 for *D* and 0.26 for *D*^+^. The null distribution for *D* (red) is multi-modal at the tails with the tails (-1 and 1) accounting for 43.2% of the values of *D*. The null distribution of *D*^+^ (blue) is centered around its average of zero. **(B)** False positive rates for *D* (red) and *D*^+^ (blue) of null distribution. The p-value in the x-axis is used to set a significance threshold to get a false positive rate in the y-axis. *D* has a false positive rate of 43.2% with p-values less than 0.43. The false positive rate of *D*^+^ is similar to the corresponding p-values up until p-value> = 0.94.

We ran msprime simulations described in the Methods section titled “Simulations to benchmark using n = 1 for all populations” using the parameters shown in Fig 2 without an instance of admixture at T_GF_ to construct a null distribution for *D* and *D*^+^ by sampling a genome from each population and computing *D* and *D*^+^ in 50 kb non-overlapping windows. We take the significance threshold values for *D* and *D*^+^ from their respective null distributions. For a p-value of 0.05, we get a signal of gene flow from P_3_ to P_2_ from the significance thresholds defined at the top 2.5% values from the null distribution of *D* and *D*^+^ (see Fig 4). Undefined values (denominator divided by 0) of *D* or *D*^+^ where no informative sites were present in the window were dropped.

When we sample a single lineage (n = 1) from each population, an introgressed window is defined as a window that has at least 10% of bases in the window overlapping with introgressed tracts from the chromosome sampled from P_2_. A 50 kb window would then have at least 5 kb bases that are introgressed from P_3_ for the sampled chromosome from P_2_. Windows that have an overlap with introgressed tracts but that are less than 10% of the bases in the window are dropped. Most of the windows have an overlap of at least 50% of the window with introgressed tracts (S2 Fig). When the sample size is more than 1, we have to redefine what is an introgressed window, and compute *D* and *D*^+^ using our frequency-based definitions.

**Fig 5:**
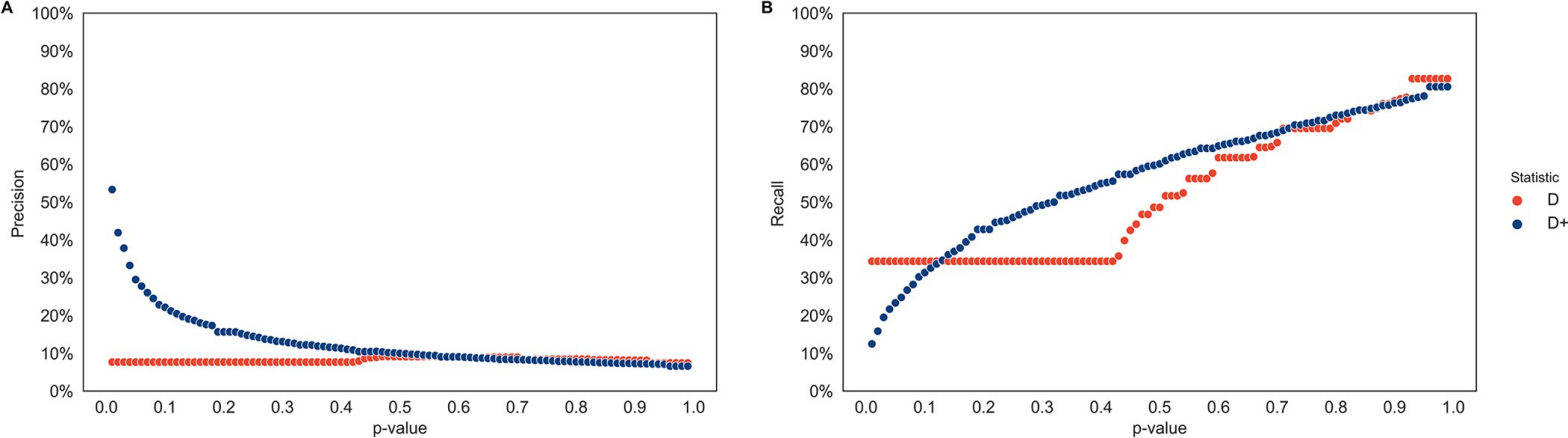
Precision and recall of *D* and *D*^+^ in simulations. The Precision-Recall of *D* and *D*^+^ for simulations with an admixture proportion of 3. *D* (red) and *D*^+^ (blue) were computed in non-overlapping 50 kb windows of 100 simulations of a 20 MB genome from each population with an admixture proportion of 3% (*f* = 0.03). (A) Precision and (B) recall are shown as a function of the p-value (0.01–1) used to get a significant threshold value of *D* and *D*^+^.

To compute recall, true positives are introgressed windows that are statistically significant, while the false negatives are introgressed windows that are not statistically significant. The false positives for the simulated data are windows that have no introgressed bases but are statistically significant. Precision measures the probability of a window truly being introgressed given that its *D*^+^ value is statistically significant. Precision is the percentage of true positives out of the sum of true positives and false positives, or 1 –false discovery rate. Recall measures how many of the introgressed windows are statistically significant and is the percentage of true positives out of the sum of true positives and false negatives. Here, false positive rate is the percentage of false positives from windows without introgression (false positives and true negatives; see Figs 4 and 5).

We also compute recall when the sample size is bigger than one. However, in this case, it will be harder to detect windows with introgressed tracts at low frequency in the recipient population. Therefore, in simulations where we sample n = 200 chromosomes from P_2_, we redefine what is an introgressed window so that two conditions need to be true. First, a window needs to contain at least one introgressed tract that survives in at least 10% of the 200 chromosomes from P_2_. Second, the length of the tract (or the sum of the tract lengths if more than one tract in a window pass the first condition) is at least 10% of the window (which is 10% of 50 kb or 5kb). For an example see S3 Fig. In S10A Fig shows recall for *D*, *D*^+^ and *d*_*f*_ as a function of the proportion of introgression. In S10B Fig, the proportion of introgression is set to f = 10% and recall is computed as a function of the required tract-frequency in P_2_ within a window (first condition necessary to define a window as introgressed).

### Testing violations of a strict molecular clock

*D*^+^ assumes a strict molecular clock such that all populations have the same mutation rate. When there is no gene flow, we can assume an equal number of ABBA and BABA sites, as well as an equal number of BAAA and ABAA sites. To test violations to this assumption we ran msprime simulations with n = 1 chromosome for P_1_, P_2_ and P_3_ where we increase the mutation rate of either P_1_ or P_2_ by increasing all of the divergence times T_P2_, T_P3_ and T_GF_ by T_P2_ in the model depicted in Fig 2. For example, to double the mutation rate of P_1_, we increase all divergence times by T_P2_ and sample P_1_ at time t = 0 and sample P_2_ at T_P2_. P_3_ is sampled right after the modified T_GF_ since P_3_ is an archaic population.

We ran 100 msprime simulations using n = 1 chromosome for P_1_, P_2_ and P_3_ under the new divergence times with no introgression and with introgression (f = 3%). The performance of *D*^+^ can be calculated using the null distribution with the new divergence times to assess statistical significance. Introgressed windows are windows where at least 10% of the bases in the 50 kb window are introgressed tracts for the haplotype from P_2_. We considered four cases: 1) P_1_ with a mutation rate double the mutation rate of P_2_, 2) P_1_ with a mutation rate ten times the mutation rate of P_2_, 3) P_2_ with a mutation rate double the mutation rate of P_1_ and 4) P_2_ with a mutation rate ten times the mutation rate of P_1_.

### Application of *D*^+^ in modern-day humans

To evaluate the performance of *D*^+^ at identifying introgressed regions in empirical data we apply *D*^+^ to previously detected regions of Neanderthal introgression in modern-day humans. We assume that introgressed segments inferred in [7] using the Altai Neanderthal genome [27] are the true introgressed segments. From the 1000 Genomes Project [28] we used an individual from the YRI (Yoruba in Ibadan, Nigeria) population for P_1_ and an individual from the GBR (British from England and Scotland) population for P_2_. P_3_ is the Altai Neanderthal genome [27]. The ancestral allele of each position was taken from the ancestral allele listed in the 1000 Genome Project. For the GBR individual we used a Neanderthal introgression map including all the haplotypes inferred to be Neanderthal with a probability > 90% in [7]. We calculated *D* and *D*^+^ in non-overlapping 50 kb windows using one autosomal chromosome of each individual from all three populations, discarding the first and last window of each chromosome. Note that here we assume that the phasing of the GBR individual is perfect, so we are able to compute two *D*^+^ and two *D* values for each window in the genome. However, we use the same chromosome in the YRI individual to compute the two *D*^+^ values for the GBR individual. Since phase is unavailable for the Neanderthal genome, we randomly sampled one of the Neanderthal alleles at each site. Each window had two *D* and *D*^+^ values, one for each autosomal chromosome of the sampled GBR individual.

**Fig 6:**
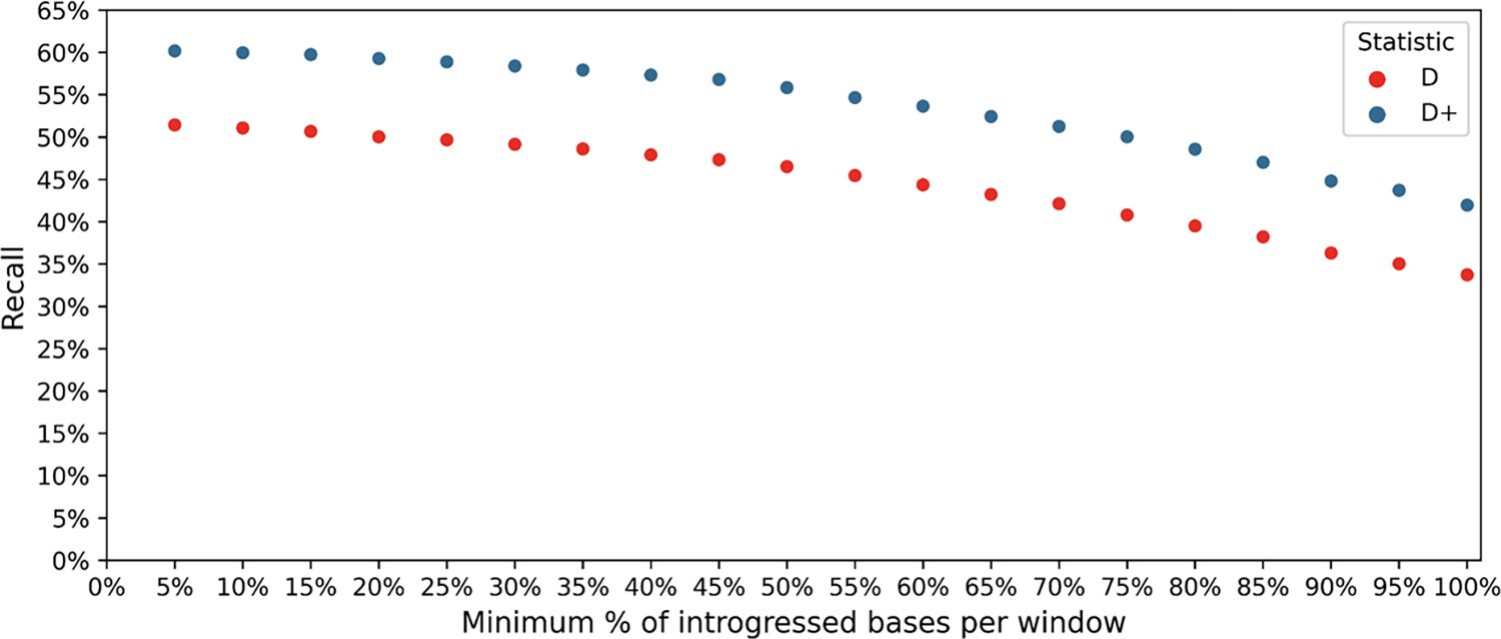
Recall of *D* and *D*^+^ in human data. The recall of *D* and *D*^+^ in non-overlapping 50 kb windows. Windows overlap with Neandertal introgression maps [7] from 5% to 100%. The populations are as follows: P_1_: YRI, P_2_: GBR, P_3_: Altai Neandertal, P_4_: Ancestral Alleles. Data for humans from 1000 Genomes Project [28] and data for Altai Neandertal from [27].

To find significance thresholds for the empirical data, we use all of the *D* and *D*^+^ values from all of our windows (two per window) to build the empirical distributions for *D* and *D*^+^. If the maximum of the two *D* (or *D*^+^) values in a window is in the top 2.5% of *D* and *D*^+^ values for the empirical distribution, then it will be called statistically significant. To compute recall, we need to define a true introgressed window that will be called a true positive or false negative based on the empirical distribution of *D* and *D*^+^ values. We defined a true introgressed window as a window with a set minimum percentage of bases that overlap with a Neanderthal introgressed segment (inferred in [7]). We used different values for the minimum percentage of bases needed to overlap with the Neanderthal segment for a window to be called introgressed. Using the empirical distribution, we call a window “introgressed” (or a true positive) when the maximum of its two *D*^+^ values are statistically significant (i.e. values are in the top 2.5% of the distribution) and it has at least a pre-defined percentage of overlap with a Neanderthal introgressed segment (e.g. 5% to 100% in intervals of 5%, x-axis of Fig 6). Recall was then calculated by dividing the number of true positives by the total number of windows defined as true introgressed windows (based on having at least a pre-defined percentage of overlap with a Neanderthal introgressed segment). We assume that the introgression maps capture true positives or a subset of them; however, we cannot assume that regions not included in the introgression maps are true negatives. Therefore, we do not assess false positives or precision.

We also took computed recall mimicking the empirical approach in simulated data where we know what the ground truth is. Specifically, in our simulations we sampled one individual (n = 2 chromosomes) from P_1_, P_2_ and P_3_ and computed *D* and *D*^+^ for each chromosome from the individual in P_2_, and also obtained the maximum *D* and *D*^+^ value per window. In the simulated data, we only considered one value (10%) for the minimum percentage of overlap between a window and an introgressed tract. We computed precision and recall using all the *D* and *D*^+^ values from all the windows as the null distribution.

Finally, we assess how recall is affected when phasing is unavailable. In this case, we use the same YRI, GBR and Neanderthal individuals and randomly sample a haplotype (n = 1 for all 3 populations) at every position to create a single haploid genome for each population. We partitioned the haploid genomes in non-overlapping 50 kb windows and compute *D*^+^ values for each window. We do this experiment 100 times and compute recall for each experiment.

### Application of *D*^+^ in *Heliconius* butterflies

*D* was applied to *Heliconius* butterflies and found to have high variance in areas of low nucleotide diversity [25]. To assess whether *D*^+^ reduces variance in these areas of low nucleotide diversity we recreated Fig 3 from [25] using the same *Heliconius* genome data from [29]. They show values of *D* as a function of nucleotide diversity π for P_2_ (the recipient population) in non-overlapping regions of 5 kb. Only biallelic alleles were used. *D* was computed using derived allele frequencies and we also use the frequencies from the four populations to compute *D*^+^ (Eq 2).

${\hat{f}}_{d}$ [25] and *d*_*f*_ [24] were also computed for the 5 kb non-overlapping windows. ${\hat{f}}_{d}$ was only applied to windows where *D* is positive. The equation for ${\hat{f}}_{d}$ written in terms of derived allele frequencies with ${\hat{p}}_{iD}$ as the maximum of ${\hat{p}}_{i2}$ and ${\hat{p}}_{i3}$ is

$${\hat{f}}_{d}=\frac{\sum_{i=1}^{L}\left(\left(1-{\hat{p}}_{i1}){\hat{p}}_{i2}{\hat{p}}_{i3}(1-{\hat{p}}_{i4}\right))-({\hat{p}}_{i1}(1-{\hat{p}}_{i2}){\hat{p}}_{i3}(1-{\hat{p}}_{i4})\right)}{\sum_{i=1}^{L}\left(\left(1-{\hat{p}}_{i1}){\hat{p}}_{iD}{\hat{p}}_{iD}(1-{\hat{p}}_{i4}\right))-({\hat{p}}_{i1}(1-{\hat{p}}_{iD}){\hat{p}}_{iD}(1-{\hat{p}}_{i4})\right)}$$

*d*_*f*_ incorporates BBAA sites where only P_1_ and P_2_ share a derived allele. The equation for *d*_*f*_ in terms of allele frequencies is

$$d_{f}=\frac{\sum_{i=1}^{L}\left(\left(1-{\hat{p}}_{i1}){\hat{p}}_{i2}{\hat{p}}_{i3}(1-{\hat{p}}_{i4}\right))-({\hat{p}}_{i1}(1-{\hat{p}}_{i2}){\hat{p}}_{i3}(1-{\hat{p}}_{i4})\right)}{\sum_{i=1}^{L}\left(\left(1-{\hat{p}}_{i1}){\hat{p}}_{i2}{\hat{p}}_{i3}(1-{\hat{p}}_{i4})+{\hat{p}}_{i1}{\hat{p}}_{i2}(1-{\hat{p}}_{i3})(1-{\hat{p}}_{i4}\right))+(\left({\hat{p}}_{i1}(1-{\hat{p}}_{i2}){\hat{p}}_{i3}(1-{\hat{p}}_{i4}))+{\hat{p}}_{i1}{\hat{p}}_{i2}(1-{\hat{p}}_{i3})(1-{\hat{p}}_{i4}\right)\right)}$$

Four samples were used, one each from *H*. *melpomene aglaope* (P_1_), the recipient population *H*.*m*. *amaryllis* (P_2_), the donor population *H*. *timareta thelxinoe* (P_3_). The outgroup (P_4_) consisted of a sample from species in the silvaniform clade including *H*. *hecale*, *H*. *ethilla*, *H*. *paradalinus sergestus* and *H*. *pardalinus ssp*. *nov*. The ancestral state of an allele was determined by the outgroup if the allele was fixed within the outgroup. Otherwise, it was the major allele of all four populations. The wing pattern loci *HmB* and *HmYb* are defined in [25]. Code was adapted from [25] with details in GitHub repository (https://github.com/LeslyLopezFang/Dplus).

Similar to the method to find recall described in “Calculating precision, recall and false positive rate in simulated human data.”, we calculate recall for *D*, *D*^+^, ${\hat{f}}_{d}$, *d*_*f*_ and *D*_*ancestral*_. The windows that overlap the *HmB* and *HmYb* loci are considered introgressed windows. Recall here is the number of these introgressed windows that the introgression statistic identifies as introgressed. Each window has one value for each statistic, and we find the statistical thresholds for each statistic by finding the top 2.5% value of the distribution.

## Results

### Theoretical results

The expectation for the values of *D* and *D*^+^ is dependent on the lengths of the branches that produce each site pattern. T_ABBA_ is the length of the branch starting from the time of the most recent common ancestor of P_2_ and P_3_ until that lineage coalesces with P_1_ (which happens in the ancestral population P_123_ under the instantaneous admixture model). The average length of the T_ABBA_ branch increases with the migration rate (Fig 3). A mutation on this branch produces an ABBA site pattern. T_BABA_ is then the length of the branch from the time of the most recent common ancestor of P_1_ and P_3_ until that lineage coalesces with P_2_. T_BAAA_ and T_ABAA_ are the external branches of P_1_ and P_2_, respectively. When there is no gene flow, the average length of the external branches of P_1_ or P_2_ are equal. With gene flow between P_2_ and P_3_, the external branch of P_1_ will be longer than the external branch of P_2_; therefore, the expectation of T_BAAA_ increases with the admixture proportion *f*.

The analytical and theoretical expectation of T_ABBA_, T_BABA_, T_BAAA_ and T_ABAA_ are shown in Fig 3. The theoretical expectation of each branch takes into account all scenarios that could produce each site pattern, including gene flow and no gene flow (S1 Appendix). The simulated expected branch lengths approximate the theoretical expected branch lengths at all the admixture proportions (*f*) calculated. When there is no admixture, the number of ABBA sites is equal to the number of BABA sites as any sharing of derived alleles between P_3_ and P_2_ (or P_3_ and P_1_) is due to incomplete lineage sorting. In the case of ancestral sharing and under a model of no admixture, the number of BAAA sites and ABAA sites will be equal because we assume equal mutation rates in P_1_ and P_2_.

For all values of migration between P_2_ and P_3_, the expected branch lengths that can lead to a BAAA (T_BAAA_) or a ABAA (T_ABAA_) site are always greater than the expected branch lengths that can lead to an ABBA (T_ABBA_) or BABA site (T_BABA_). Therefore, if we assume a constant mutation rate, we expect to see more ABAA sites than BABA sites and more BAAA sites than ABBA sites. In Fig 3, assuming a constant mutation rate multiplied with the analytical and simulated expected branch lengths, there are 5–6 times more BAAA and ABAA sites than ABBA and BABA sites.

Interestingly, our theoretical results also show that even though the number of BAAA and ABAA is higher (than ABBA or BABA), the difference between T_BAAA_ and T_ABAA_ (T_BAAA_—T_ABAA_) is equal to the difference (T_ABBA_—T_BABA_). Therefore, for all admixture proportions between P_2_ and P_3_, the expected difference of BAAA and ABAA sites (BAAA—ABAA) is equal to the expected difference of ABBA and BABA sites (ABBA—BABA). These observations suggest that leveraging ancestral shared variation can be informative about introgression and provides justification for defining *D*^+^ which leverages both ancestral and derived allele sharing to maximize the number of informative sites used in a genomic window. This increase in informative sites can provide greater predictive accuracy for detecting local gene flow.

### *D* has a high false positive rate in small genomic windows

We calculated *D* and *D*^+^ for 50 kb windows on simulated genomes following the demography in Fig 2 with no admixture event at T_GF_ to get the null distribution of *D* and *D*^+^ (Fig 4A). The null distribution of *D* is a multimodal distribution with large peaks at the tails as well as zero. The average of *D* is 0 with a standard deviation of 0.74. The tails (*D* = 1 and *D* = −1) account for 43.2% of the distribution. These peaks at the tails cause a high false positive rate of 43.2% for *D* at p-values less than 0.43 (Fig 4B) because the significance threshold for *D* is 1 or -1. Therefore, we have low power to assess statistically significant values of *D*. In contrast *D*^+^ has a null distribution centered on zero. The average of *D*^+^ is 0 and the standard deviation is 0.26. The null distribution is much narrower than the null distribution of *D* and does not have peaks at the tails. As expected, the false positive rate of *D*^+^ approximates the p-value set to find significant values of *D*^+^ up until a significance threshold approaches 0 for high p-values (p-values > = 0.94) (Fig 4A).

### *D*^+^ has better precision than *D* in simulated data

We calculated precision and recall for 50 kb windows of 100 simulations with a 20 MB simulated genome shown in Fig 5 following the demography in Fig 2. Undefined values were dropped (see Methods) so more windows were analyzed for *D*^+^ than *D* because *D* had more undefined values. While precision measures the accuracy of windows giving a signal of gene flow from P_3_ to P_2_ through statistical significance, recall measures how many introgressed windows the statistic can detect without considering false positives. We obtained precision and recall for p-values from 0.01–1 (Fig 5). Each p-value has a corresponding significant threshold value from the null distribution in Fig 4A in which values of *D* or *D*^+^ greater than the threshold are statistically significant. For realistic p-values (i.e. p-values 0.01, 0.02, 0.03, 0.04 and 0.05), *D*^+^ has better precision than *D*; At these realistic p-values, precision for *D*^+^ ranges from 29.48% to 53.30% and the precision of *D* is 7.65% (Fig 5A). For these p-values, *D* has better recall than *D*^+^ (Fig 5B) with recall for *D*^+^ ranging from 12.46% to 23.33% and recall for *D* equaling 34.33%. For *D*, precision and recall are the same (7.65% and 34.33% p-values < 0.43, because the *D* value is 1 since the null distribution is multimodal with peaks at the tails (Fig 4A). It should be noted that these results are robust to different window thresholds—i.e., introgressed tracts covering at least 5%, 10%, and 25% of a 50kb window (S24 Fig). Notably, when we consider a more complex human demography (see S5 Fig), the recall and precision is 58.71% and 37.34% at a p-value of 0.05 (S6 Fig). One of the reasons the recall is higher under the more complex model is because the effective population size of Neanderthals is smaller. This means that the Neanderthal sequenced used to compute the *D* statistics is more closely related to the actual Neanderthals that introgressed into modern humans.

To see how changes in the admixture proportion, mutation and recombination rate affect the performance of *D*^+^, we also simulated under different admixture proportions, recombination rates and mutation rates. We find that precision is sensitive to the admixture proportion (S7 Fig). For a p-value of 0.05, a higher admixture proportion of f = 5% increases precision by 16% and an admixture proportion of f = 10% increases precision by 36% in comparison to an admixture proportion of f = 3% shown in Fig 5. In contrast, decreasing the admixture proportion from f = 3% to 2% decreases the precision by 6%. Recall is less sensitive to the admixture proportion than precision with the biggest change happening for f = 10% with an increase of 4%. The recall and precision for other p-values is shown in S7 Fig.

Changing the mutation rate affects the number of informative sites per window and changing the recombination rate affects the length of the introgressed segments and the number of windows that count as introgressed. We show precision and recall for different p-values in S8 and S9 Figs for a larger and smaller mutation rate and recombination rate. For a p-value of 0.05, increasing the mutation rate by a factor of two increases precision by approximately 4% and increases recall by approximately 2% (S8 Fig compared to Fig 5). In contrast, when the mutation rate is decreased by a factor of two, the precision and recall drop by 4% and 7% respectively (S8 Fig). Decreasing the recombination rate decreases the number of windows that are introgressed. These windows contain more overlap with an introgressed segment since the introgressed segments are longer. By contrast, increasing the recombination rate by a factor of 2 almost doubled the number of windows that are introgressed; however, the introgressed segments within a window are shorter in length. When the recombination rate decreases by a factor of two, recall increases by 2% (S9B Fig) and precision increases by 7% (S9A Fig). When the recombination rate is doubled, recall decreased by 7% (S9E Fig) and precision increases by 2% (S9D Fig). For p-values of 0.01, 0.02, 0.03, 0.04 and 0.05, the false positive rate for a model with no gene flow remains relatively constant as the admixture proportion, mutation rate or recombination rate change (S7C, S7F, S7I, S8C, S8F, S9C and S9F Figs).

*D*^+^ can also be applied to sample sizes greater than one using derived allele frequencies with Eq 2. For simulations with n>1, we use admixture proportions greater than 3% (see Methods). We compute *D*^+^ for simulations with admixture proportions of 10%, 20%, 30%, 40% and 50% and computed recall (S10A Fig). Note that we have a different definition of what an introgressed window is, which we explained in the Methods section titled “Calculating precision, recall and false positive rate in simulated human data” and an example is provided in S3 Fig. For a p-value of 0.05 *D*^+^ has higher recall than *D* for all admixture proportions. The recall of *D*^+^ increases as the admixture proportion increases. As two conditions need to be met to call a window introgressed (see Methods), we considered relaxing the first assumption involving the frequency of the introgressed tract in the recipient population (P_2_). When we change the frequency of the introgressed tract(s) in P_2_, recall increases as the frequency of the tract increases (see S10B and S10C Fig). Furthermore, we ran additional simulations with a realistic admixture proportion of 3% and computed precision and recall for a different set of chromosome and window thresholds (see S1 Text section “Comparing performance of D and D+ for different chromosome and window thresholds”). We find that for realistic p-values D+ will always have a higher precision and recall than D, which demonstrates that for a realistic admixture proportion, increased number of sampled chromosomes, and for all pairwise possibilities of chromosome and window thresholds D+ consistently outperforms D for detecting signals of introgression at a local scale (S25 and S26 Figs).

### *D*^+^ performs well under moderate violations of the molecular clock

Under a strict molecular clock, we expect the number of ABBA and BABA sites and the number of BAAA and ABAA sites to be equal under the null model with no gene flow. To assess the performance of *D*^+^ when the mutation rate of P_1_ and P_2_ are not equal, we increased the mutation rate for P_1_ and P_2_ in comparison to each other by a factor of 2 and 10.

When P_1_ has a higher mutation rate the amount of BAAA sites is greater than the amount of ABAA sites under no admixture (S11A Fig). This skews the distribution and average of *D*^+^ towards 1, a signal of introgression from P_3_ into P_2_. Similarly, when P_2_ has a larger mutation rate, the amount of ABAA sites is greater than the amount of BAAA sites under the model of no admixture and makes the average of *D*^+^ negative instead of 0, indicating gene flow from P_3_ to P_1_ (S11B Fig).

For a p-value of 0.05, precision of *D*^+^ is 31.60% when the mutation rate of P_1_ is double the mutation rate of P_2_ and precision is 37.22% when the mutation rate of P_2_ is double the mutation rate of P_1_ (S12 Fig). In a more extreme scenario, increasing the mutation rate of either P_1_ or P_2_ by a factor of ten decreases the precision in comparison to either P_1_ or P_2_ having double the mutation rate. At a p-value of 0.05, precision decreases to 23.95% when P_1_ has a higher mutation rate than P_2_ and precision decreases to 29.36% when P_2_ has a higher mutation rate than P_1_ (S12 Fig). In general, these results suggest that precision is only mildly affected by the differences in mutation rate between P_1_ and P_2_.

When P_2_ is the population with double the mutation rate, recall of *D*^+^ is 18.35%, and 23.77% when the mutation rate of P_1_ is doubled (S12 Fig). When P_1_ is ten times the mutation rate of P_2_, recall is 16.32%, and 10.00% when P_2_ is ten times the mutation rate of P_1_ (S12 Fig). It makes sense that recall is worse when the mutation rate is higher in P_2_ since this increases the number of ABAA sites, so the difference (BAAA-ABAA) is smaller which means the signal of introgression is smaller.

### *D*^+^ identifies Neanderthal introgressed regions in modern-day humans

To investigate the behavior of *D*^+^ in real data, we applied *D*^+^ to modern-day humans [28] and an Altai Neanderthal [27] to find if signals of gene flow corresponded to previously identified Neanderthal introgressed regions. Unlike simulated data, in real human genomes we do not know the ground truth, and to compare the performance of *D* and *D*^+^, we assumed that the Neanderthal introgressed regions from [7] were the truth. We calculated *D* and *D*^+^ windows for the two phased chromosomes of a single GBR individual from [28] to compute the recall of *D* and *D*^+^ (Fig 6). Since the 50 kb windows will sometimes only partially contain an introgressed segment, we defined a window as introgressed if the window had a minimum percentage of bases overlapping with an introgressed segment (see Methods). Statistical significance was computed using the genome-wide distribution of *D*^+^ values (or *D* values) as the null distribution. Recall is the number of these “true” introgressed windows that were called statistically significant over the total number of introgressed windows (see Methods).

Fig 6 shows that recall for *D*^+^ was consistently better than *D* as a function of the minimum percentage of introgressed bases in a window. The recall decreases as the minimum overlap between an inferred introgressed segment and a window increases. This happens because the number of introgressed windows used to calculate recall decreases when we increase the amount of overlap to call a window introgressed. We note that for this analysis the *D*^+^ value assigned for each window was the maximum of the two *D*^+^ values for each of the two phased chromosomes in the GBR individual. We tested this method of choosing the maximum of *D*^+^ values per window on simulated data (under the demographic history in Fig 2) and computed the precision and recall for this scenario (see S13 Fig). We found that for a p-value of 0.05 the recall is 23.56% and precision is 44.69%. Taking the maximum of *D*^+^ values per window did not affect the recall in comparison to the recall of *D*^+^ when only one chromosome from P_2_ is used to compute *D*^+^ (see Fig 5). By comparison, the corresponding recall in the empirical data is around 59% (recall when the point on the x-axis is 10% in Fig 6) which is similar to the recall under a more complex human demographic model (S6 Fig). The complex human demographic model (shown in S5 Fig) includes a smaller effective population size for the Neanderthal population.

In the previous analysis, we assumed that the chromosomes of the GBR individual are perfectly phased. However, as the phasing is inferred, there could be phasing errors and/or often phased chromosomes may not be available. When phasing is unavailable, studies randomly sample a single allele to create a haploid genome (e.g. as in the Neanderthal genome). We tested how this works in both real and simulated data. We implemented this approach with the individuals we used for Fig 6 and compute recall 100 times and find that recall for a p-value of 0.05 is on average 24.07% (see S14 Fig), similar to the recall in the simulated data, with recall ranging from 22.39–25.67% for all 100 runs. Therefore, we recommend that when the phase is not available or is uncertain, the user can randomly sample a chromosome from each individual as is often done in ancient DNA studies.

### *D*^+^ can detect introgression events in regions of low nucleotide diversity

One of the main reasons the *D* statistic is not useful for detecting introgression in small regions of the genome is that the variance of *D* is high in areas of low nucleotide diversity [25]. To address this [25] proposed ${\hat{f}}_{d}$ as an alternative approach to quantify and detect introgression in small genomic regions. The numerator of ${\hat{f}}_{d}$ is in the same form as that of *D*; however, the denominator of ${\hat{f}}_{d}$ replaces the derived allele frequency of P_2_ and P_3_ with the maximum derived allele frequency of P_2_ and P_3_. This leads to ${\hat{f}}_{d}$ having a lower variance in areas of low nucleotide diversity, thus reducing spurious results in comparison to *D*. Like ${\hat{f}}_{d}$, *d*_*f*_ is also designed to quantify the admixture proportion of small genomic regions [24]. The approach in *d*_*f*_ is to incorporate BBAA sites as fewer sites with this pattern are expected when introgression occurs between P_2_ and P_3_ or between P_1_ and P_3_.

**Fig 7:**
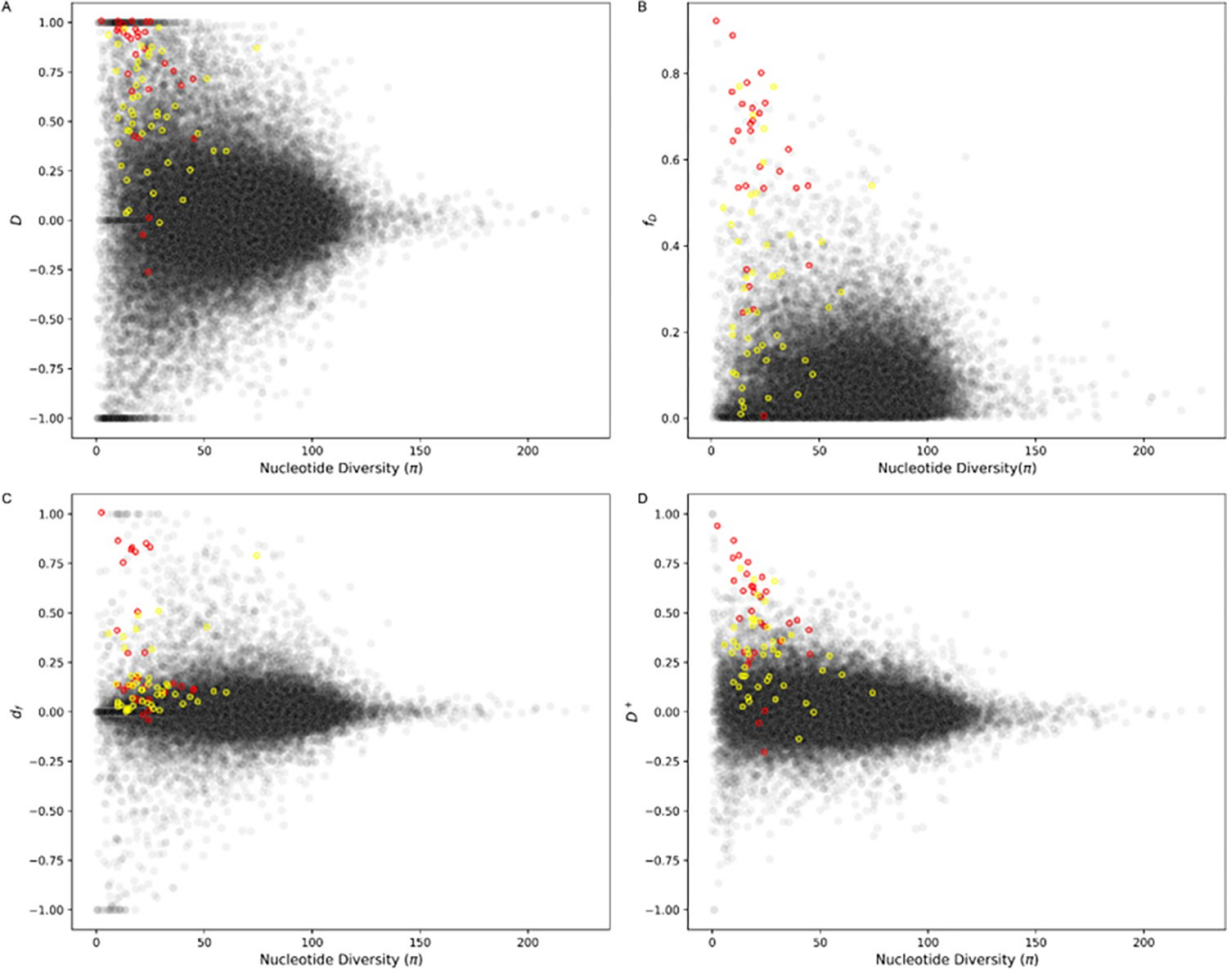
Application of *D*, ${\hat{f}}_{d}$, *d*_*f*_, and *D*^+^ in *Heliconius* butterfly. (A) *D*, (B) ${\hat{f}}_{d}$, (C) *d*_*f*_ and (D) *D*^+^ as a function of nucleotide diversity in P_2_ in non-overlapping 5 kb windows. P_1_: *H*. *melpomene aglaope*, P_2_: *H*. *melpomene amaryllis*, P_3_: *H*. *timareta thelxinoe*, P_4_: *H*. *hecale*, *H*. *ethilla*, *H*. *paradalinus sergestus* and *H*. *pardalinus ssp*. *nov*. from the silvaniform clade. Red and yellow circles correspond to windows with introgressed loci HmB and HmYb, respectively. Methods follow Fig 3 from [25] with *Helicionius* genome data from [29].

Both ${\hat{f}}_{d}$, *d*_*f*_ are estimates of the admixture proportion while *D* and *D*^+^ are used to detect and not quantify introgression. To compare *D*^+^ to ${\hat{f}}_{d}$ and *d*_*f*_ we used the same *Heliconius* genome data from [29]. *Heliconius* butterflies have strong evidence for both genome-wide and adaptive introgression between species, including mimicry loci for wing patterns [14,29,30]. We use these data to compute these statistics in windows as a function of nucleotide diversity, since the relationship between *D* and nucleotide diversity observed in [29] inspired the developments of new statistics to detect and quantify introgression in small windows of the genome. For the four populations, we use *H*. *melpomene aglaope* as P_1_, *H*. *melpomene amaryllis* as P_2_, *H*. *timareta thelxinoe* as P_3_ and the *H*. *hecale*, *H*. *ethilla*, *H*. *paradalinus sergestus* and *H*. *pardalinus ssp*. *nov*. species in the silvaniform clade as the outgroup (P_4_). We compute nucleotide diversity π, ${\hat{f}}_{d}$, *d*_*f*_, *D* and *D*^+^ in non-overlapping 5 kb windows. Windows from the candidate introgressed loci responsible for the red wing pattern (*HmB*) and the yellow and white wing pattern (*HmYb*) are shown in red and yellow, respectively, in Fig 7. We find similar results as [25]; *D* has a high variance and a wide distribution in regions of low nucleotide diversity (Fig 7A). As nucleotide diversity increases the distribution of *D* narrows. ${\hat{f}}_{d}$ reduces the high variance of values in areas of low nucleotide diversity (Fig 7B). *d*_*f*_ also reduces variance with most of the *d*_*f*_ values centered around zero, including windows with the *HmB* and *HmYb* loci (Fig 7C). *D*^+^ has smaller variance with fewer outliers than *D* and similar variance to *d*_*f*_ (Fig 7D). *D*^+^ detects candidate regions of introgression, including windows not detected by D (see S1 Table). Many of the windows that *D*^+^ detects as introgressed correspond to the candidate regions for introgression that have been previously suggested in *Heliconius* butterflies and are associated with wing patterning (red and yellow points in Fig 7). In fact, *D*^+^ detects approximately 68.4% of these candidate introgressed windows. In comparison, *D* detects 52.6%, *d*_*f*_ detects 27.6% and ${\hat{f}}_{d}$ detects 63.1%. We also computed *D*_*ancestral*_ which only uses the ancestral shared patterns (ABAA and BAAA), and it detects 63.2% of the windows (S15 Fig), suggesting that using the ancestral site patterns alone is better behaved than the *D* statistic, which shows the utility of using ancestral shared variation.

## Discussion

Multiple studies have found that introgression plays an important evolutionary role as it introduces new genetic variation in a population that can be targeted by natural selection; this is an accelerated process of accumulating new alleles compared to a *de novo* mutation process. Therefore, detecting which regions of the genome exhibit signatures of introgression is an important step to evaluate its relative contribution to evolution. To date, Patterson’s *D* statistic is the most widely used metric for detection of introgression genome wide. While *D* works well at detecting introgression at the genome-wide scale, some studies have shown that *D* might not be the best choice to detect introgression in small regions of the genome. In this paper, we define a new statistic, *D*^+^, that leverages both sites with shared ancestral and sites with shared derived alleles to improve detection of introgression in small genomic windows.

First, we use coalescent theory to understand this statistic’s theoretical properties and derive the expectation of *D*^+^ as a function of gene flow. We show that the expected counts of BAAA sites and ABAA sites are equal under a model of no introgression. As the proportion of admixture increases, one of these two site patterns increases, implying that BAAA and ABAA sites are informative to detect introgression. Interestingly, our theoretical results also show that the expected difference in counts of BAAA and ABAA sites equals the expected difference of ABBA and BABA sites (Fig 3). However, in general there are more BAAA and ABAA sites than ABBA and BABA sites. *D*^+^ is more conservative than *D* with a smaller expectation and variance than *D* in small genomic windows (Figs 4 and S1). As a result, *D*^+^ has less false positives than *D*, likely because *D*^+^ includes more informative sites (Fig 4). Therefore, *D*^+^ also has better precision than *D* in simulated data under the Neanderthal admixture model presented in Fig 2 (Fig 5A) and under more realistic human demography models (S6 Fig).

We also apply *D*^+^ to detect Neanderthal introgression in a non-African individual. Unlike simulations, in real data we do not know the ground truth. Therefore, we evaluated *D*^+^ by asking: if we assume the existing inferred maps [7] are the truth, how often do we call a window introgressed when it completely or partially contains an introgressed segment? Under these assumptions, we find that recall is around 59% (see Fig 6) which is similar to the simulation results under a complex demographic history (see S6 Fig). Overall, our simulations and empirical data suggest that *D*^+^ has statistical properties that make it more stable than *D* at detecting introgression in small genomic windows and provides an alternative method to detect introgression.

We also evaluated the performance of *D*^+^ as a function of the admixture proportion, mutation rate and recombination rate, and for the values considered, our simulations show that the impact on recall or on precision is not too high (S7, S8 and S9 Figs). *D*^+^ is also robust to realistic violations of the molecular clock under a human demography (S12 Fig). We consider cases when the mutation rate differed (between P1 and P2) by a factor of two or ten. Differences in mutation rate can mimic signatures of introgression, and do affect the performance of the statistic (see S11 and S12 Figs). We find that a higher mutation rate in P_2_ than P_1_ would hinder the performance of *D*^+^ more than a higher mutation rate in P_1_ than P_2_ because this produces more ABAA sites. We note, however, that in real data, it will be rare to observe differences in the mutation rate that are this extreme.

Another factor that could affect the performance of *D*^+^ is differences in sequencing error in P1 vs. P2. To evaluate this, we simulated differences in sequencing errors across lineages, and they do not appear to have a large impact on the overall positive rate of *D*^+^ (S16, S17 and S18 Figs; S1 Text). Thus, when *D*^+^ is calculated on a window level we conclude that *D*^+^ is robust to differential sequencing errors. Finally, we considered whether *D*^+^ can distinguish incomplete lineage sorting (ILS) from introgression at the local level. Using the whole genome, we know that ILS leads to an equal number of BAAA and ABAA sites, so at the genome level these sites cancel out. Our simulation results show that *D*^+^ is better than *D* at distinguishing ILS at the local level (see S19–S22 Figs; S1 Text).

There are other methods such as ${\hat{f}}_{d}$ [25] and *d*_*f*_ [24] that have been derived from Patterson’s *D* to quantify the admixture proportion, *f*, in small genomic regions. ${\hat{f}}_{d}$ leverages ABBA and BABA sites, *d*_*f*_ leverages ABBA, BABA and BBAA sites, and *D*^+^ leverages ABBA, BABA, BAAA and ABAA sites. To compare with these methods, we ran simulations following the demography depicted in Fig 2 and computed *D*, *d*_*f*_ and *D*^+^ and found that the performance of *D*^+^ and *d*_*f*_ are comparable (S10 Fig). We also applied them to a *Heliconius* butterflies data set, and we found that similarly to ${\hat{f}}_{d}$ and *d*_*f*_, the variance of *D*^+^ is reduced in regions of low nucleotide diversity. This suggests that like ${\hat{f}}_{d}$ and *d*_*f*_, *D*^+^ will also not lead to a high number of false positives, especially in regions of low nucleotide diversity. Indeed, we find that many of the regions with a signal of introgression from windows contain previously identified candidate introgressed loci. All these statistics have both shared and distinct aspects in how they leverage genetic patterns, and future studies might focus on integration of these approaches to improve the detection and quantification of introgression. Specifically, probabilistic models that incorporate these site patterns as features might provide better inferences of introgression. We recognize that all these statistics have been benchmarked to detect or quantify introgression under very specific and simple demographic scenarios that may not closely reflect the true demographic histories of actual species or populations. Future studies that compare and contrast how different statistics that detect and quantify introgression [24,25,31–34] behave under more complex demographic scenarios and under different evolutionary time scales will help characterize the behavior of these statistics and expand our understanding of the power and limitations of each method.

In summary, we have shown that ancestral shared variation between a donor and recipient population is influenced by the introgression proportion. Leveraging this ancestral sharing in the *D* statistic through *D*^+^ can improve inferences of local introgression. *D*^+^ be applied locally and on a genome-wide scale (S23 Fig). Our results suggest that shared ancestral variation is informative for detecting introgression on both local and global scales, and might also be useful for deriving new estimators of the proportion of introgression that may help address how pervasive introgression is across the tree of life. Beyond their utility to detect introgression, there is evidence that archaic introgression may have re-introduced ancestral alleles with regulatory effects in humans [35], pointing to the importance of studying ancestral shared variation. We expect that more studies will reveal the effects and consequences of re-introducing ancestral variation, and that leveraging ancestral information may be informative on ghost admixture events from uncharacterized ghost populations [27].

## Supporting information

S1 Fig Branches of T_ABBA_, T_BABA_, T_BAAA_ and T_ABAA_. The branch lengths of T_ABBA_ (blue), T_BABA_ (yellow), T_BAAA_ (green) and T_ABAA_ (red) correspond to branches where a mutation on that branch would lead to an ABBA, BABA, BAAA and ABAA site, respectively.
(TIFF)

S2 Fig Number of introgressed bases in a 50 kb window for simulated genomes following demographic model in Fig 2. The histogram depicts the distribution of the number of base pairs within a 50 kb window that have a genealogical history of introgression.
(TIFF)

S3 Fig Example of an introgressed window when we simulate with n>1. Vertical dash lines represent the boundaries of a 50kb window. Solid horizontal lines represent chromosomes and blue rectangles represent introgressed tracts. Two conditions need to be true. First, we ask, is there at least one introgressed tract (depicted by the blue rectangles) that is present at frequency of at least 10% in P_2_ (at least present in two chromosomes out of 12 in this example). In this example there are 3 tracts where that condition is met. We then add up the lengths of those introgressed tracts and ask: is the sum of lengths of the tracts at least 10% of 50 kb? In this example, the sum of the lengths of the three tracts is 15 kb. In this example both conditions are met, so this would be defined as an introgressed window.
(TIFF)

S4 Fig Theoretical and analytical expectations of *D* and *D*+. Analytical (lines) and simulated (dots) expectation of *D* (red) and *D+* (blue) as a function of the admixture proportion (*f*) of 0, 0.01, 0.02, 0.05, 0.1, 0.2, 0.5 and 1. The simulated expectations of *D* and *D+* concur with the analytical expectations. The expectation of *D* and *D+* are both zero when there is no gene flow and both expectations increase as *f* increases.
(TIFF)

S5 Fig Realistic demographic model of human evolution modified from Ragsdale and Gravel (2019). The model in Ragsdale and Gravel (2019) has continuous bidirectional migration but this modified model has three discrete pulses. The first pulse of unidirectional migration is from the Neanderthal population to the ancestral population of CEU and CHB. The second and third pulse of unidirectional migration is from the Neanderthal population to the CHB population and from the Neanderthal population to the CEU population. Solid arrows represent population divergences and dashed arrows represent gene flow events.
(TIFF)

S6 Fig Performance of *D* and *D*+ on simulated haplotypes under a modified model from Ragsdale and Gravel (2019). (A) Precision, (B) recall and (C) false positive of *D* and *D+* calculated in 50 kb windows for 100 msprime simulations of 20 MB genomes with n = 1 for P_1_, P_2_ and P_3_ following the model in [36] modified to include unidirectional pulses of migration, described in [37]. The false positive rate is under a model with no introgression.
(TIFF)

S7 Fig Performance of *D* and *D+* on simulated haplotypes with different admixture proportions. (A,D,G) Precision, (B,E,H) recall and (C,F,I) false positive rate for *D* and *D+* calculated in 50 kb windows for 100 msprime simulations of 20 MB genomes with n = 1 for P_1_, P_2_ and P_3_ following the demography in Fig 2 with admixture proportions: (A-C) *f = 2%*, (D-F) *f = 5%* and (G-I) *f = 10%*. The false positive rate is under a model with no introgression.
(TIFF)

S8 Fig Performance *D* and *D+* on simulated haplotypes with different mutation rates. (A, D) Precision, (B, E) recall and (C, F) false positive rate for *D* and *D+* calculated in 50 kb windows for 100 msprime simulations of 20 MB genomes with n = 1 for P_1_, P_2_ and P_3_ following the demography in Fig 2 with (A-C) half the default mutation rate and (D-F) twice the default mutation rate. The default mutation rate is 1.5* 10^−8^ per bp per generation. The false positive rate is under a model with no introgression.
(TIFF)

S9 Fig Performance *of D* and *D+* on simulated haplotypes with different recombination rates. (A,D) Precision, (B,E) recall and (C,F) false positive rate for *D+* calculated in 50 kb windows for 100 msprime simulations of 20 MB genomes with n = 1 for P_1_, P_2_ and P_3_ following the demography in Fig 2 with (A-C) half the default recombination rate and (D-F) twice the default recombination rate. The default recombination rate is 10^−8^ per bp per generation. The false positive rate is under a model with no introgression.
(TIFF)

S10 Fig Performance of *D*, *df* and *D+* on simulated genomes using derived frequencies. The recall (p-value of 0.05) of *D*, *df* and *D+* calculated using derived frequencies in 50 kb windows of 100 msprime simulations of 20 MB genomes following the demography in Fig 2. We sampled n = 200 chromosomes for P_1_ and P_2_ and n = 2 chromosomes for P_3_. (A) Recall as a function of *f = 10%*, *20%*, *30%*, *40%* and *50%*. Here we defined an introgressed window as a window where two conditions are true: 1) at least one tract is present in at least 20 chromosomes in P_2_ (equivalent to a frequency of 10% in P_2_) and 2) the sum of the introgressed tracts lengths (that are present within the window at frequency of 10% in P_2_) is at least 5 kb of the 50 kb window. This is definition described in S3 Fig. (B) Recall when we set f = 10% and we relax the second condition described in part A. Here we allow the tracts to have frequencies in P_2_ of 0.5%, 5%, 10%, 15%, or 20% (x-axis). (C) Same as B but setting f = 30%.
(TIFF)

S11 Fig Distribution of *D+* when P_1_ and P_2_ have different mutation rates. (A) P_1_ has double the mutation rate of P_2_ and (B) P_2_ has double the mutation rate of P_1_. *D+* is calculated in 50 kb windows for 100 msprime simulations of 20 MB genomes with n = 1 for P_1_, P_2_ and P_3_ following the demography in Fig 2 with divergence rates increased by a factor of T_P2_ to increase mutation rate of (A) P_1_ or (B) P_2_.
(TIFF)

S12 Fig Performance of *D+* when P_1_ and P_2_ have different mutation rates. (A, C) Precision, (B, D) recall for *D+* calculated in 50 kb windows for 100 msprime simulations of 20 MB genomes with n = 1 for P_1_, P_2_ and P_3_ following the demography in Fig 2 with divergence rates increased by a factor of T_P2_ to increase mutation rate of (A,B) P_1_ or (C,D) P_2_. The mutation rate of (A,B) P_1_ is twice and ten times the mutation rate of P_2_ and (C,D) P_2_ is twice and ten times the mutation rate of P_1_. The default mutation rate for P_1_ and P_2_ is 1.5* 10^−8^ per bp per generation.
(TIFF)

S13 Fig Precision and Recall for simulated data using the maximum *D* and *D+* value per window. (A) Precision and (B) recall are computed for 50 kb windows of 100 20 MB simulated genomes with n = 1 individual from P_1_, P_2_ and P_3_ using the maximum *D* and *D+* value per window when *D* and *D+* are calculated for both chromosomes of the P_2_ individual and the same chromosome for the P_1_ and P_3_ individual.
(TIFF)

S14 Fig Recall of *D*^+^ for human empirical data assuming the phase is unknown. *D+* was calculated in 50 kb windows by randomly sampling a haplotype at every position for an individual from the African (YRI), non-African (GBR), and archaic (Neanderthal) population, where this process was replicated 100 times. For each replicate recall was computed as the number of these “true” introgressed windows that were called statistically significant over the total number of introgressed windows, where the “true” introgressed windows were determined by the introgression maps from [7].
(TIFF)

S15 Fig Application of *Dancestral* in *Heliconius* butterfly. *Dancestral* as a function of nucleotide diversity in P_2_ in non-overlapping 5 kb windows. P_1_: *H*. *melpomene aglaope*, P_2_: *H*. *melpomene amaryllis*, P_3_: *H*. *timareta thelxinoe*, P_4_: *H*. *hecale*, *H*. *ethilla*, *H*. *paradalinus sergestus* and *H*. *pardalinus ssp*. *nov*. from the silvaniform clade. Red and yellow circles correspond to windows with candidate introgressed loci *HmB* and *HmYb*, respectively. Methods follow Fig 3 from [25] with *Helicionius* genome data from [29].
(TIFF)

S16 Fig Distributions of synthetic sequencing errors in 50kb windows. Assuming a sequencing error rate of 0.001 and a genome size of 3Gb, 100 replication simulations were conducted for (column A), sequencing errors in only P_1_ (column B), sequencing errors in only P_2_ (column C), and sequencing errors in both P_1_ and P_2_ (column D). The distributions represent the observed number of sequencing errors in 50 kb windows with the mean and standard deviation denoted.
(TIFF)

S17 Fig Distributions of *D* and *D*^+^ for simulations without introgression. Using the demographic model described in Fig 2 without introgression *D* (blue) and *D+* (green) were calculated in 50 kb windows from 100 replicate simulations with no sequencing errors (column A), sequencing errors in only P_1_ (column B), sequencing errors in only P_2_ (column C), and sequencing errors in both P_1_ and P_2_ (column D), where we simulated a genome size of 100 Mb and assumed a sequencing error rate of 1e-4.
(TIFF)

S18 Fig False positive rates for *D* and *D*^+^. The p-value in the x-axis is used to set a significance threshold to get a false positive rate in the y-axis of null distributions following the demographic model in Fig 2 without introgression, where *D* (blue) and *D+* (green) were calculated in 50 kb windows from 100 replicate simulations with no sequencing errors (column A), sequencing errors in only P_1_ (column B), sequencing errors in only P_2_ (column C), and sequencing errors in both P_1_ and P_2_ (column D), where we simulated a genome size of 100 Mb and assumed a sequencing error rate of 1e-4.
(TIFF)

S19 Fig Distribution of *D* values conditioned on coalescent histories. Based on 100,000 replicate simulations of unliked loci with a mutation rate of 1.5e-8 the distributions of *D* are shown for coalescent histories of ILS with no introgression (top row), and coalescent histories of ILS (middle row) vs introgression (bottom row) given and admixture proportion of 0.03 and the IUA demographic model described in the methods section for loci of size 10kb (column A), 20kb (column B), 30kb (column C), 40kb (column D), and 50kb (column E).
(TIFF)

S20 Fig Quantile-Quantile plot corresponding to *D* value distributions conditioned on coalescent histories. Based on 100,000 replicate simulations of unliked loci with a mutation rate of 1.5e-8 the observed quantiles (y-axis), theoretical quantiles (x-axis), and the line of best fit with the associated coefficient of determination are shown for coalescent histories of ILS with no introgression (top row), and coalescent histories of ILS (middle row) vs introgression (bottom row) given and admixture proportion of 0.03 and the IUA demographic model described in the methods section for loci of size 10kb (column A), 20kb (column B), 30kb (column C), 40kb (column D), and 50kb (column E) to assess if the observed *D* distributions are normally distributed around mean 0 and scaled by the observed standard deviation of each respective distribution.
(TIFF)

S21 Fig Distribution of *D*+ values conditioned on coalescent histories. Based on 100,000 replicate simulations of unliked loci with a mutation rate of 1.5e-8 the distributions of *D+* are shown for coalescent histories of ILS with no introgression (top row), and coalescent histories of ILS (middle row) vs introgression (bottom row) given and admixture proportion of 0.03 and the IUA demographic model described in the methods section for loci of size 10kb (column A), 20kb (column B), 30kb (column C), 40kb (column D), and 50kb (column E).
(TIFF)

S22 Fig Quantile-Quantile plot corresponding to *D*+ value distributions conditioned on coalescent histories. Based on 100,000 replicate simulations of unliked loci with a mutation rate of 1.5e-8 the observed quantiles (y-axis), theoretical quantiles (x-axis), and the line of best fit with the associated coefficient of determination are shown for coalescent histories of ILS with no introgression (top row), and coalescent histories of ILS (middle row) vs introgression (bottom row) given and admixture proportion of 0.03 and the IUA demographic model described in the methods section for loci of size 10kb (column A), 20kb (column B), 30kb (column C), 40kb (column D), and 50kb (column E) to assess if the observed *D*+ distributions are normally distributed around mean 0 and scaled by the observed standard deviation of each respective distribution.
(TIFF)

S23 Fig Genome-wide power of *D* and *D+* to detect introgression. Power of *D* (blue) *D+* (green) to detect introgression from 100 replicate simulations with a genome size of 100 Mb for sample sizes of n = 1 (top row) and n = 100 (bottom row) monoploid genomes from P_1_ and P_2_ under the IUA model described in the methods (column A), and a realistic model of human demographic history described in Ragsdale and Gravel 2019 for the CEU (column B) and CHB (column C) populations.
(TIFF)

S24 Fig Precision and recall of *D* and *D*^+^ in simulations with variable window thresholds. The Precision-Recall of *D* (blue) and *D*^+^ (green) were computed in non-overlapping 50 kb windows of 100 simulations of a 20 MB genome sampling a single chromosome from each focal population with an admixture proportion of 3% (*f* = 0.03). Using window thresholds—i.e., introgressed tracts covering at least 5% (A & D), 10% (B & E), and 25% (C & F) of a 50kb window. Precision and recall are shown as a function of the p-value (0.01–1) used to get a significant threshold value of *D* and *D*^+^.
(TIFF)

S25 Fig Precision and of *D* and *D*^+^ in simulations with variable chromosome window thresholds. The Precision of *D* (blue) and *D*^+^ (green) were computed in non-overlapping 50 kb windows of 100 simulations of a 20 MB genome sampling 200 chromosomes from P1 and P2 and two chromosomes from P3 with an admixture proportion of 3% (*f* = 0.03). Using all pairwise combinations of requiring introgressed tracts to be present in at least 5% (top row), 10% (middle row), and 25% (bottom row) of sampled P2 chromosomes and requiring introgressed tracts to cover at least 5% (left column), 10% (middle column), and 25% (right column) of a 50kb window. Precision is shown as a function of the p-value (0.01–1) used to get a significant threshold value of *D* and *D*^+^.
(TIFF)

S26 Fig Recall and of *D* and *D*^+^ in simulations with variable chromosome window thresholds. The Recall of *D* (blue) and *D*^+^ (green) were computed in non-overlapping 50 kb windows of 100 simulations of a 20 MB genome sampling 200 chromosomes from P1 and P2 and two chromosomes from P3 with an admixture proportion of 3% (*f* = 0.03). Using all pairwise combinations of requiring introgressed tracts to be present in at least 5% (top row), 10% (middle row), and 25% (bottom row) of sampled P2 chromosomes and requiring introgressed tracts to cover at least 5% (left column), 10% (middle column), and 25% (right column) of a 50kb window. Recall is shown as a function of the p-value (0.01–1) used to get a significant threshold value of *D* and *D*^+^.
(TIFF)

S1 Table The number of possible candidate regions of introgression in Heliconius melpomene amaryllis from Heliconius timareta thelxinoe identified by only *D*+, only *D*, and both *D* and *D*+. (XLSX)

S1 Text Description of the supplemental analyses. (DOCX)

S1 Appendix Coalescent-based derivations of BAAA, ABAA, and *D*^+^. (DOCX)
