# Supplementary figures and images for: Leveraging shared ancestral variation to detect local introgression
Source: PLoS Genet. 2024 Jan 8;20(1):e1010155. doi: 10.1371/journal.pgen.1010155 (PMC10798638; doi:10.1371/journal.pgen.1010155)

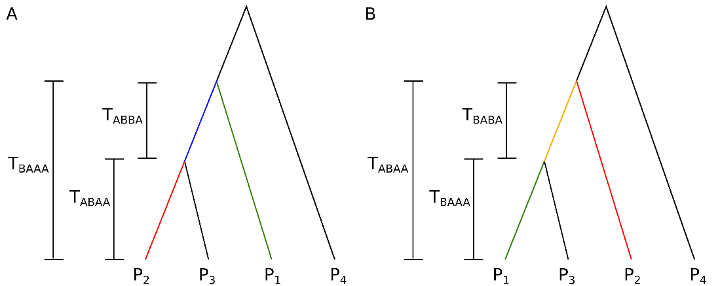

Supplement: S1 Fig — The branch lengths of TABBA (blue), TBABA (yellow), TBAAA (green) and TABAA (red) correspond to branches where a mutation on that branch would lead to an ABBA, BABA, BAAA and ABAA site, respectively. (TIFF) [file pgen.1010155.s001.tiff]

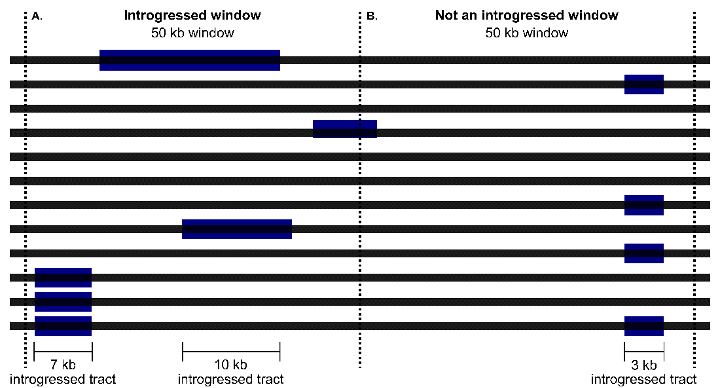

Supplement: S3 Fig — Vertical dash lines represent the boundaries of a 50kb window. Solid horizontal lines represent chromosomes and blue rectangles represent introgressed tracts. Two conditions need to be true. First, we ask, is there at least one introgressed tract (depicted by the blue rectangles) that is present at frequency of at least 10% in P2 (at least present in two chromosomes out of 12 in this example). In this example there are 3 tracts where that condition is met. We then add up the lengths of those introgressed tracts and ask: is the sum of lengths of the tracts at least 10% of 50 kb? In this example, the sum of the lengths of the three tracts is 15 kb. In this example both conditions are met, so this would be defined as an introgressed window. (TIFF) [file pgen.1010155.s003.tiff]

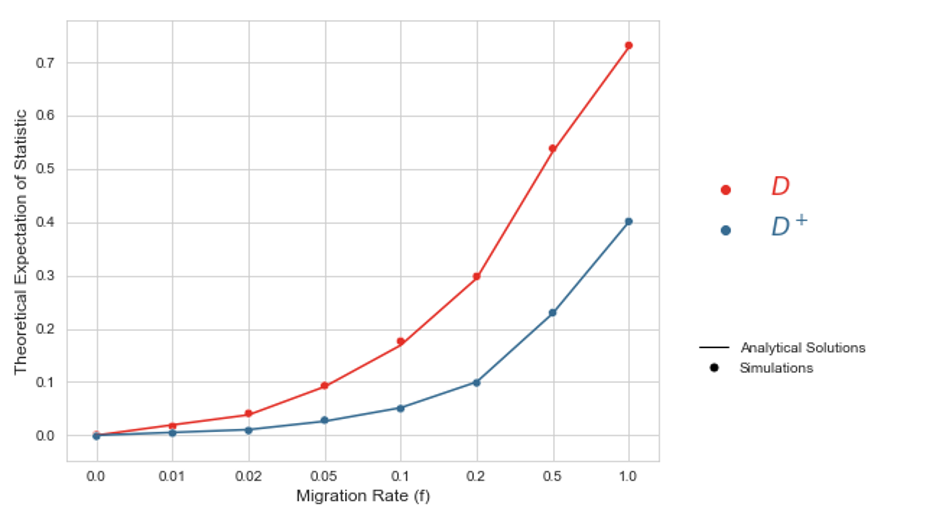

Supplement: S4 Fig — Analytical (lines) and simulated (dots) expectation of D (red) and D+ (blue) as a function of the admixture proportion (f) of 0, 0.01, 0.02, 0.05, 0.1, 0.2, 0.5 and 1. The simulated expectations of D and D+ concur with the analytical expectations. The expectation of D and D+ are both zero when there is no gene flow and both expectations increase as f increases. (TIFF) [file pgen.1010155.s004.tiff]

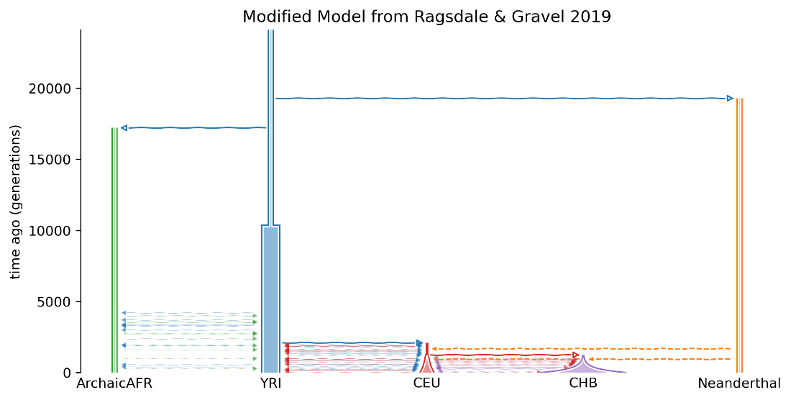

Supplement: S5 Fig — The model in Ragsdale and Gravel (2019) has continuous bidirectional migration but this modified model has three discrete pulses. The first pulse of unidirectional migration is from the Neanderthal population to the ancestral population of CEU and CHB. The second and third pulse of unidirectional migration is from the Neanderthal population to the CHB population and from the Neanderthal population to the CEU population. Solid arrows represent population divergences and dashed arrows represent gene flow events. (TIFF) [file pgen.1010155.s005.tiff]

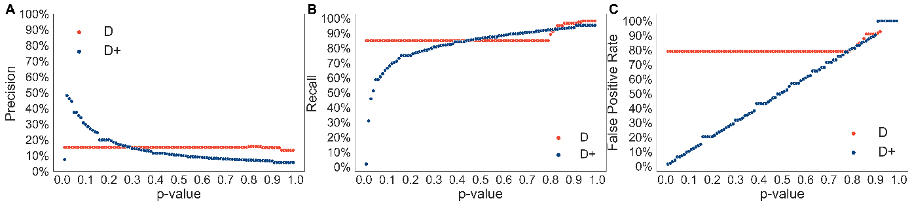

Supplement: S6 Fig — (A) Precision, (B) recall and (C) false positive of D and D+ calculated in 50 kb windows for 100 msprime simulations of 20 MB genomes with n = 1 for P1, P2 and P3 following the model in [36] modified to include unidirectional pulses of migration, described in [37]. The false positive rate is under a model with no introgression. (TIFF) [file pgen.1010155.s006.tiff]

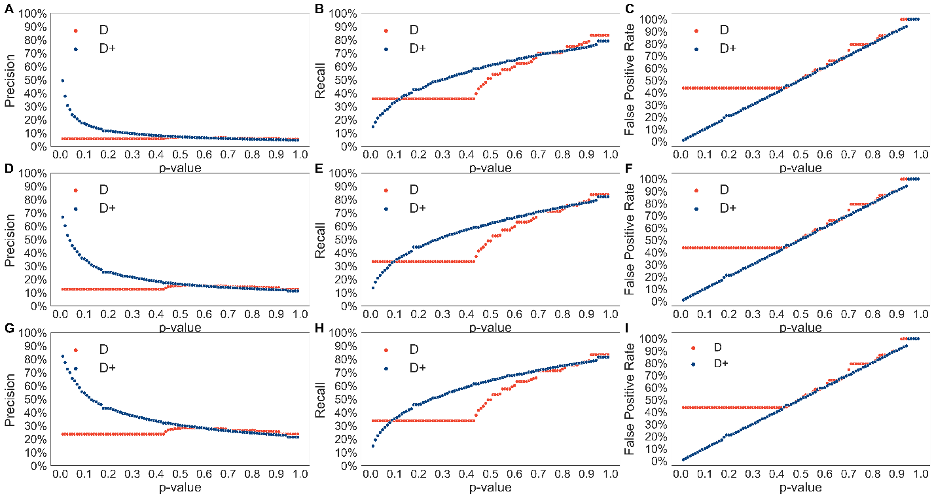

Supplement: S7 Fig — (A,D,G) Precision, (B,E,H) recall and (C,F,I) false positive rate for D and D+ calculated in 50 kb windows for 100 msprime simulations of 20 MB genomes with n = 1 for P1, P2 and P3 following the demography in Fig 2 with admixture proportions: (A-C) f = 2%, (D-F) f = 5% and (G-I) f = 10%. The false positive rate is under a model with no introgression. (TIFF) [file pgen.1010155.s007.tiff]

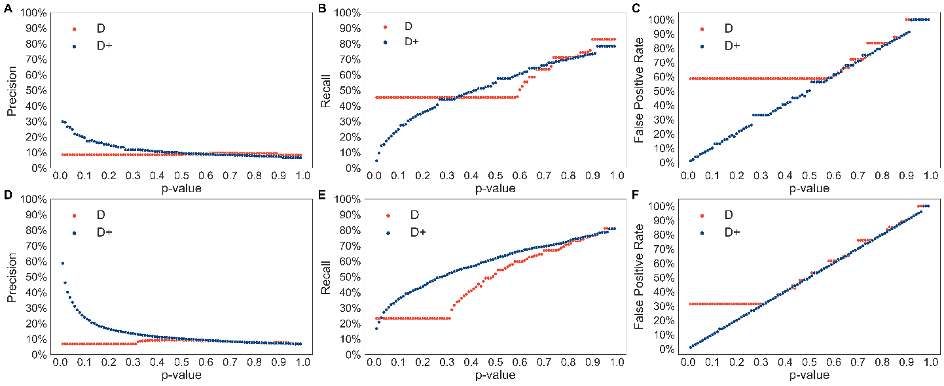

Supplement: S8 Fig — (A, D) Precision, (B, E) recall and (C, F) false positive rate for D and D+ calculated in 50 kb windows for 100 msprime simulations of 20 MB genomes with n = 1 for P1, P2 and P3 following the demography in Fig 2 with (A-C) half the default mutation rate and (D-F) twice the default mutation rate. The default mutation rate is 1.5* 10−8 per bp per generation. The false positive rate is under a model with no introgression. (TIFF) [file pgen.1010155.s008.tiff]

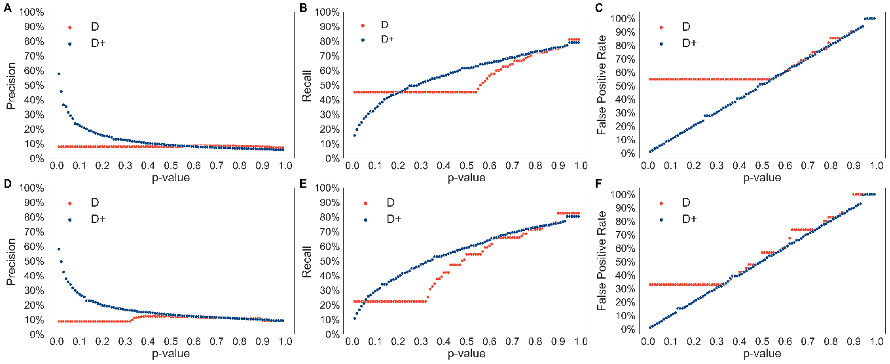

Supplement: S9 Fig — (A,D) Precision, (B,E) recall and (C,F) false positive rate for D+ calculated in 50 kb windows for 100 msprime simulations of 20 MB genomes with n = 1 for P1, P2 and P3 following the demography in Fig 2 with (A-C) half the default recombination rate and (D-F) twice the default recombination rate. The default recombination rate is 10−8 per bp per generation. The false positive rate is under a model with no introgression. (TIFF) [file pgen.1010155.s009.tiff]

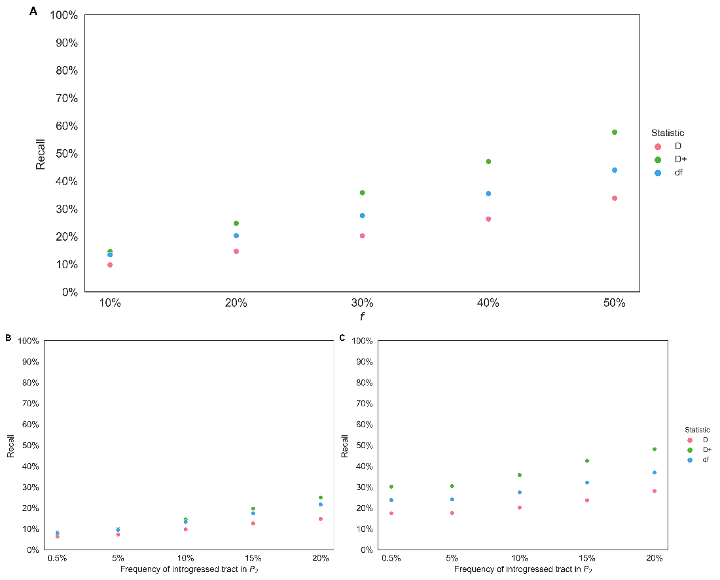

Supplement: S10 Fig — The recall (p-value of 0.05) of D, df and D+ calculated using derived frequencies in 50 kb windows of 100 msprime simulations of 20 MB genomes following the demography in Fig 2. We sampled n = 200 chromosomes for P1 and P2 and n = 2 chromosomes for P3. (A) Recall as a function of f = 10%, 20%, 30%, 40% and 50%. Here we defined an introgressed window as a window where two conditions are true: 1) at least one tract is present in at least 20 chromosomes in P2 (equivalent to a frequency of 10% in P2) and 2) the sum of the introgressed tracts lengths (that are present within the window at frequency of 10% in P2) is at least 5 kb of the 50 kb window. This is definition described in S3 Fig. (B) Recall when we set f = 10% and we relax the second condition described in part A. Here we allow the tracts to have frequencies in P2 of 0.5%, 5%, 10%, 15%, or 20% (x-axis). (C) Same as B but setting f = 30%. (TIFF) [file pgen.1010155.s010.tiff]

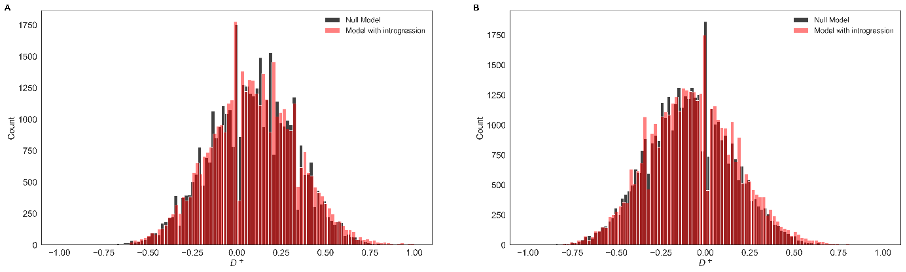

Supplement: S11 Fig — (A) P1 has double the mutation rate of P2 and (B) P2 has double the mutation rate of P1. D+ is calculated in 50 kb windows for 100 msprime simulations of 20 MB genomes with n = 1 for P1, P2 and P3 following the demography in Fig 2 with divergence rates increased by a factor of TP2 to increase mutation rate of (A) P1 or (B) P2. (TIFF) [file pgen.1010155.s011.tiff]

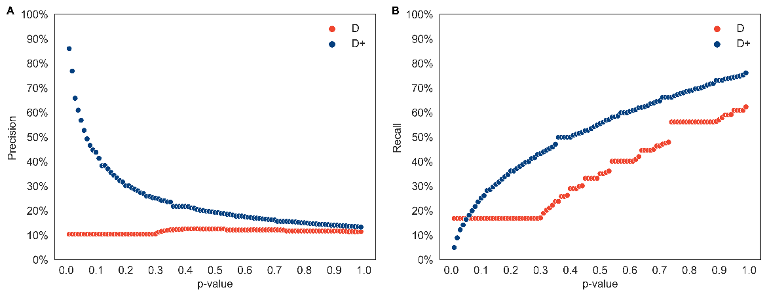

Supplement: S13 Fig — (A) Precision and (B) recall are computed for 50 kb windows of 100 20 MB simulated genomes with n = 1 individual from P1, P2 and P3 using the maximum D and D+ value per window when D and D+ are calculated for both chromosomes of the P2 individual and the same chromosome for the P1 and P3 individual. (TIFF) [file pgen.1010155.s013.tiff]

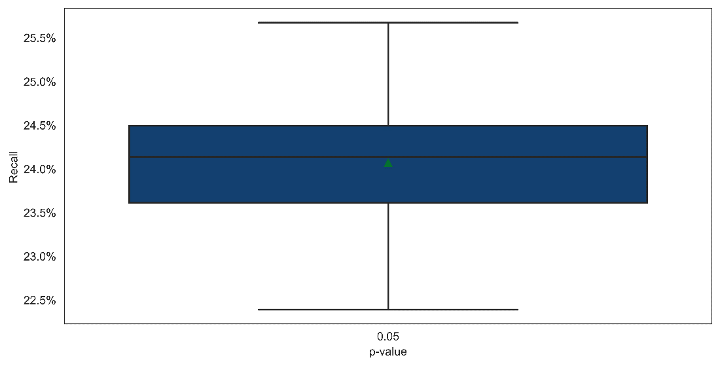

Supplement: S14 Fig — D+ was calculated in 50 kb windows by randomly sampling a haplotype at every position for an individual from the African (YRI), non-African (GBR), and archaic (Neanderthal) population, where this process was replicated 100 times. For each replicate recall was computed as the number of these “true” introgressed windows that were called statistically significant over the total number of introgressed windows, where the “true” introgressed windows were determined by the introgression maps from [7]. (TIFF) [file pgen.1010155.s014.tiff]

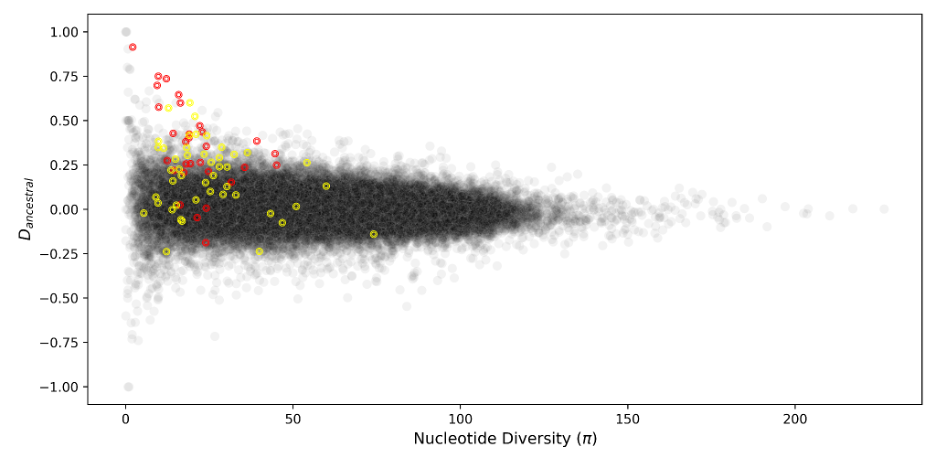

Supplement: S15 Fig — Dancestral as a function of nucleotide diversity in P2 in non-overlapping 5 kb windows. P1: H. melpomene aglaope, P2: H. melpomene amaryllis, P3: H. timareta thelxinoe, P4: H. hecale, H. ethilla, H. paradalinus sergestus and H. pardalinus ssp. nov. from the silvaniform clade. Red and yellow circles correspond to windows with candidate introgressed loci HmB and HmYb, respectively. Methods follow Fig 3 from [25] with Helicionius genome data from [29]. (TIFF) [file pgen.1010155.s015.tiff]

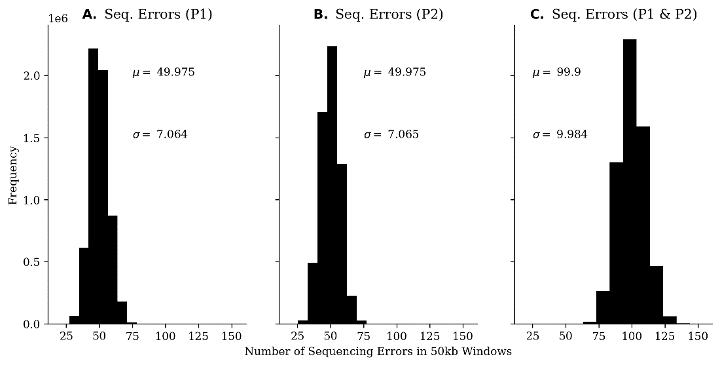

Supplement: S16 Fig — Assuming a sequencing error rate of 0.001 and a genome size of 3Gb, 100 replication simulations were conducted for (column A), sequencing errors in only P1 (column B), sequencing errors in only P2 (column C), and sequencing errors in both P1 and P2 (column D). The distributions represent the observed number of sequencing errors in 50 kb windows with the mean and standard deviation denoted. (TIFF) [file pgen.1010155.s016.tiff]

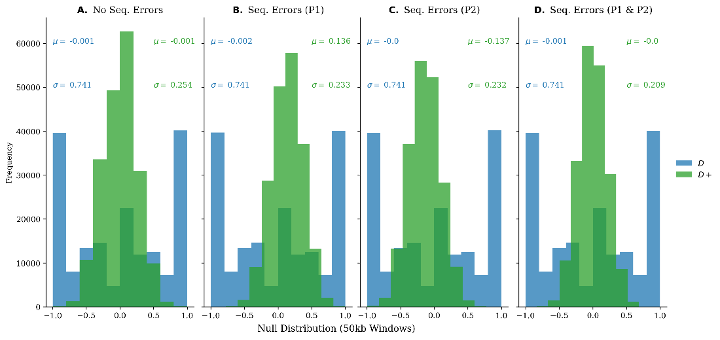

Supplement: S17 Fig — Using the demographic model described in Fig 2 without introgression D (blue) and D+ (green) were calculated in 50 kb windows from 100 replicate simulations with no sequencing errors (column A), sequencing errors in only P1 (column B), sequencing errors in only P2 (column C), and sequencing errors in both P1 and P2 (column D), where we simulated a genome size of 100 Mb and assumed a sequencing error rate of 1e-4. (TIFF) [file pgen.1010155.s017.tiff]

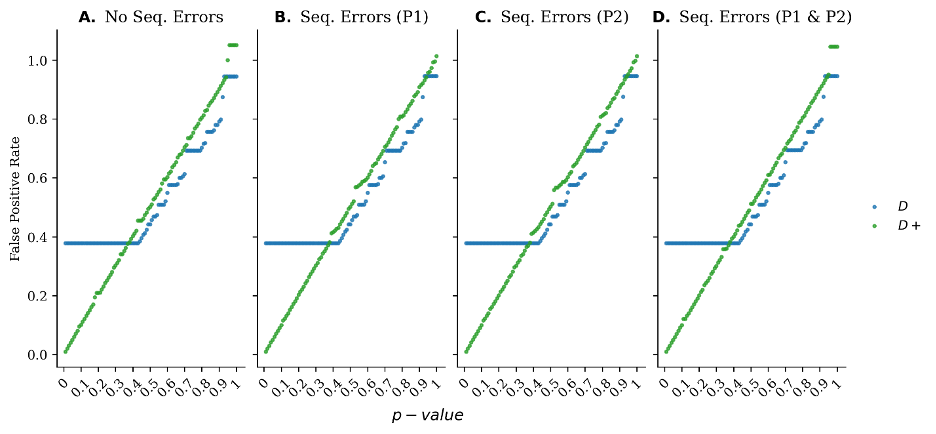

Supplement: S18 Fig — The p-value in the x-axis is used to set a significance threshold to get a false positive rate in the y-axis of null distributions following the demographic model in Fig 2 without introgression, where D (blue) and D+ (green) were calculated in 50 kb windows from 100 replicate simulations with no sequencing errors (column A), sequencing errors in only P1 (column B), sequencing errors in only P2 (column C), and sequencing errors in both P1 and P2 (column D), where we simulated a genome size of 100 Mb and assumed a sequencing error rate of 1e-4. (TIFF) [file pgen.1010155.s018.tiff]

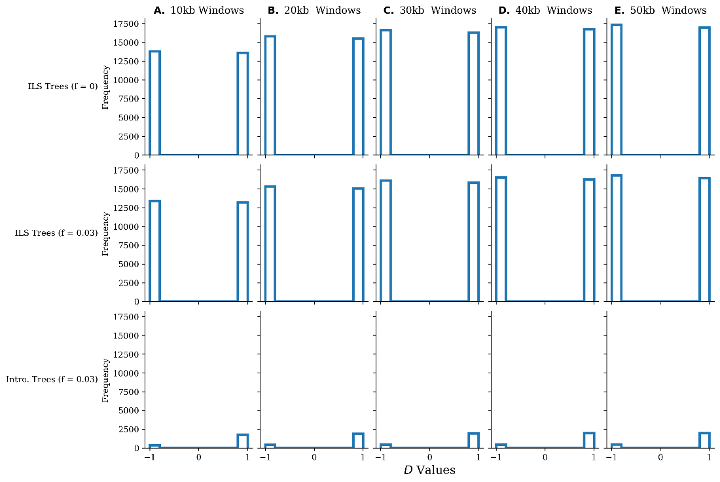

Supplement: S19 Fig — Based on 100,000 replicate simulations of unliked loci with a mutation rate of 1.5e-8 the distributions of D are shown for coalescent histories of ILS with no introgression (top row), and coalescent histories of ILS (middle row) vs introgression (bottom row) given and admixture proportion of 0.03 and the IUA demographic model described in the methods section for loci of size 10kb (column A), 20kb (column B), 30kb (column C), 40kb (column D), and 50kb (column E). (TIFF) [file pgen.1010155.s019.tiff]

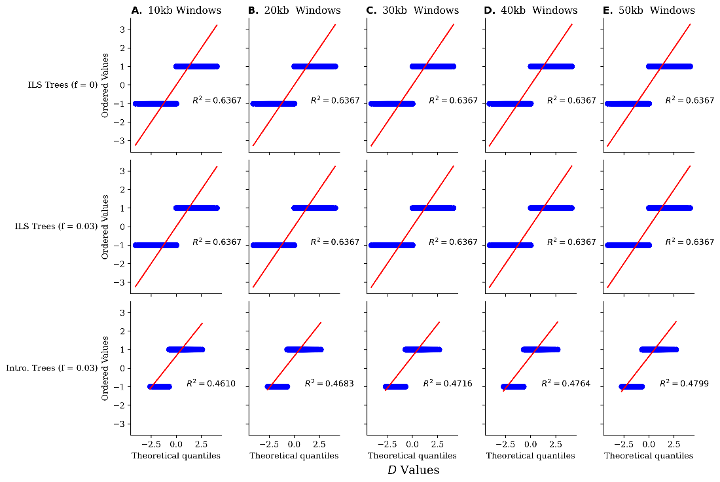

Supplement: S20 Fig — Based on 100,000 replicate simulations of unliked loci with a mutation rate of 1.5e-8 the observed quantiles (y-axis), theoretical quantiles (x-axis), and the line of best fit with the associated coefficient of determination are shown for coalescent histories of ILS with no introgression (top row), and coalescent histories of ILS (middle row) vs introgression (bottom row) given and admixture proportion of 0.03 and the IUA demographic model described in the methods section for loci of size 10kb (column A), 20kb (column B), 30kb (column C), 40kb (column D), and 50kb (column E) to assess if the observed D distributions are normally distributed around mean 0 and scaled by the observed standard deviation of each respective distribution. (TIFF) [file pgen.1010155.s020.tiff]

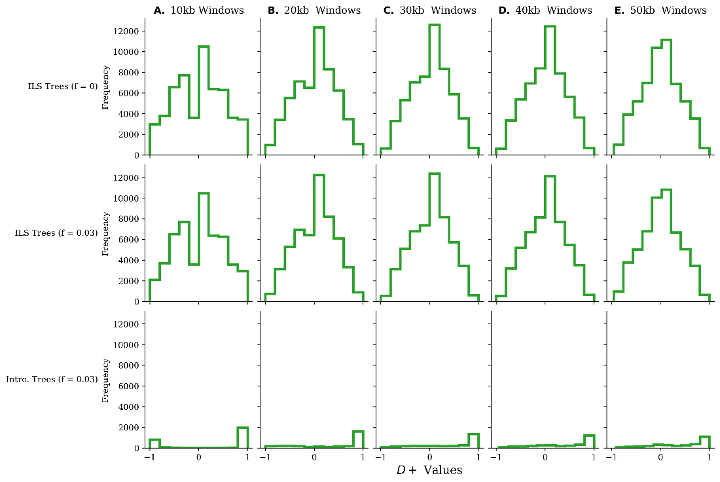

Supplement: S21 Fig — Based on 100,000 replicate simulations of unliked loci with a mutation rate of 1.5e-8 the distributions of D+ are shown for coalescent histories of ILS with no introgression (top row), and coalescent histories of ILS (middle row) vs introgression (bottom row) given and admixture proportion of 0.03 and the IUA demographic model described in the methods section for loci of size 10kb (column A), 20kb (column B), 30kb (column C), 40kb (column D), and 50kb (column E). (TIFF) [file pgen.1010155.s021.tiff]

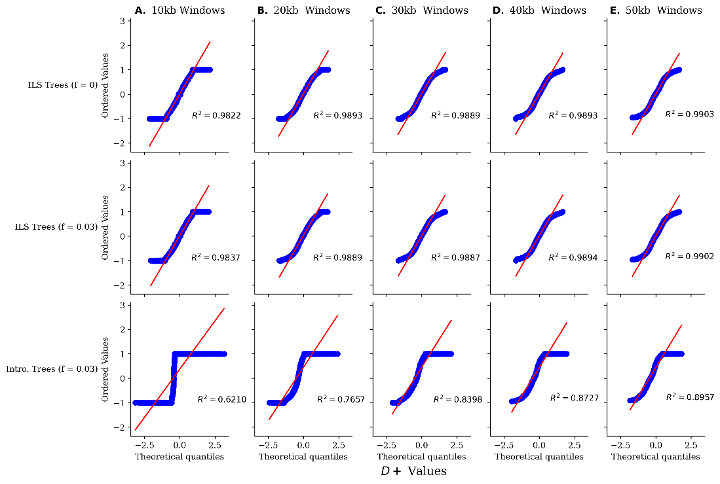

Supplement: S22 Fig — Based on 100,000 replicate simulations of unliked loci with a mutation rate of 1.5e-8 the observed quantiles (y-axis), theoretical quantiles (x-axis), and the line of best fit with the associated coefficient of determination are shown for coalescent histories of ILS with no introgression (top row), and coalescent histories of ILS (middle row) vs introgression (bottom row) given and admixture proportion of 0.03 and the IUA demographic model described in the methods section for loci of size 10kb (column A), 20kb (column B), 30kb (column C), 40kb (column D), and 50kb (column E) to assess if the observed D+ distributions are normally distributed around mean 0 and scaled by the observed standard deviation of each respective distribution. (TIFF) [file pgen.1010155.s022.tiff]

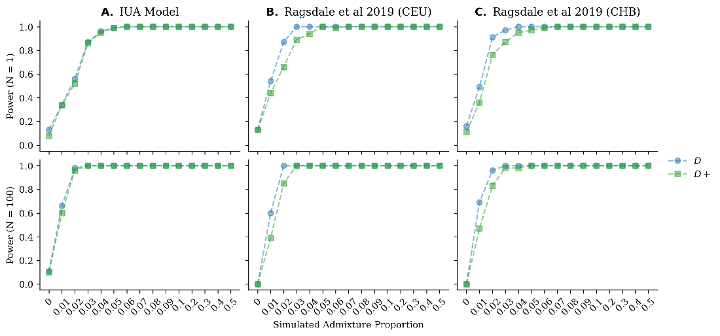

Supplement: S23 Fig — Power of D (blue) D+ (green) to detect introgression from 100 replicate simulations with a genome size of 100 Mb for sample sizes of n = 1 (top row) and n = 100 (bottom row) monoploid genomes from P1 and P2 under the IUA model described in the methods (column A), and a realistic model of human demographic history described in Ragsdale and Gravel 2019 for the CEU (column B) and CHB (column C) populations. (TIFF) [file pgen.1010155.s023.tiff]

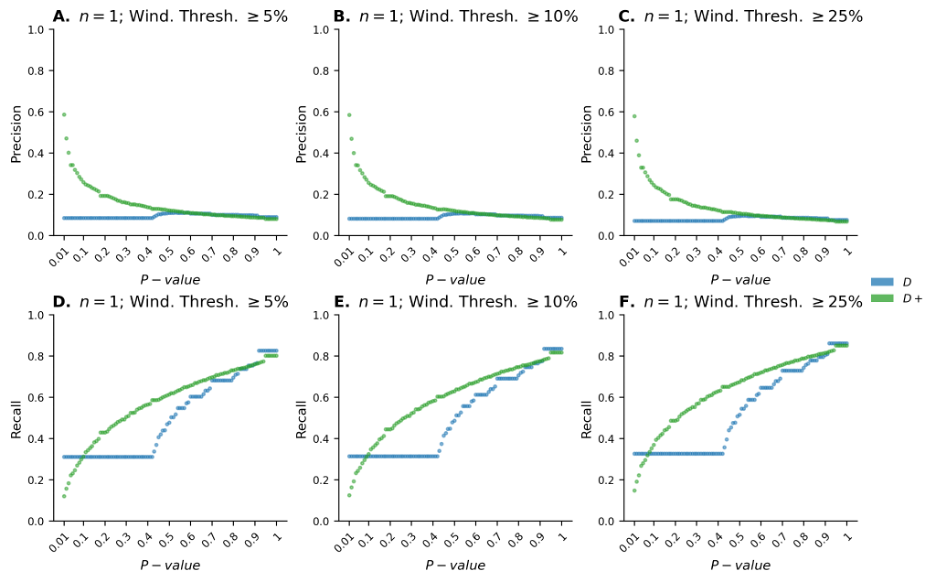

Supplement: S24 Fig — The Precision-Recall of D (blue) and D+ (green) were computed in non-overlapping 50 kb windows of 100 simulations of a 20 MB genome sampling a single chromosome from each focal population with an admixture proportion of 3% (f = 0.03). Using window thresholds—i.e., introgressed tracts covering at least 5% (A & D), 10% (B & E), and 25% (C & F) of a 50kb window. Precision and recall are shown as a function of the p-value (0.01–1) used to get a significant threshold value of D and D+. (TIFF) [file pgen.1010155.s024.tiff]

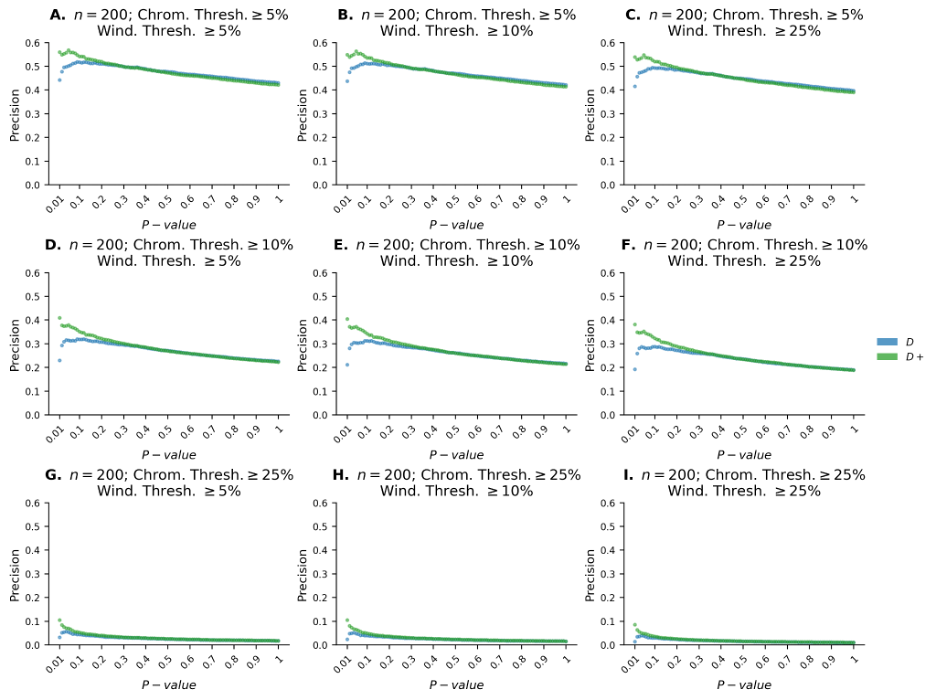

Supplement: S25 Fig — The Precision of D (blue) and D+ (green) were computed in non-overlapping 50 kb windows of 100 simulations of a 20 MB genome sampling 200 chromosomes from P1 and P2 and two chromosomes from P3 with an admixture proportion of 3% (f = 0.03). Using all pairwise combinations of requiring introgressed tracts to be present in at least 5% (top row), 10% (middle row), and 25% (bottom row) of sampled P2 chromosomes and requiring introgressed tracts to cover at least 5% (left column), 10% (middle column), and 25% (right column) of a 50kb window. Precision is shown as a function of the p-value (0.01–1) used to get a significant threshold value of D and D+. (TIFF) [file pgen.1010155.s025.tiff]

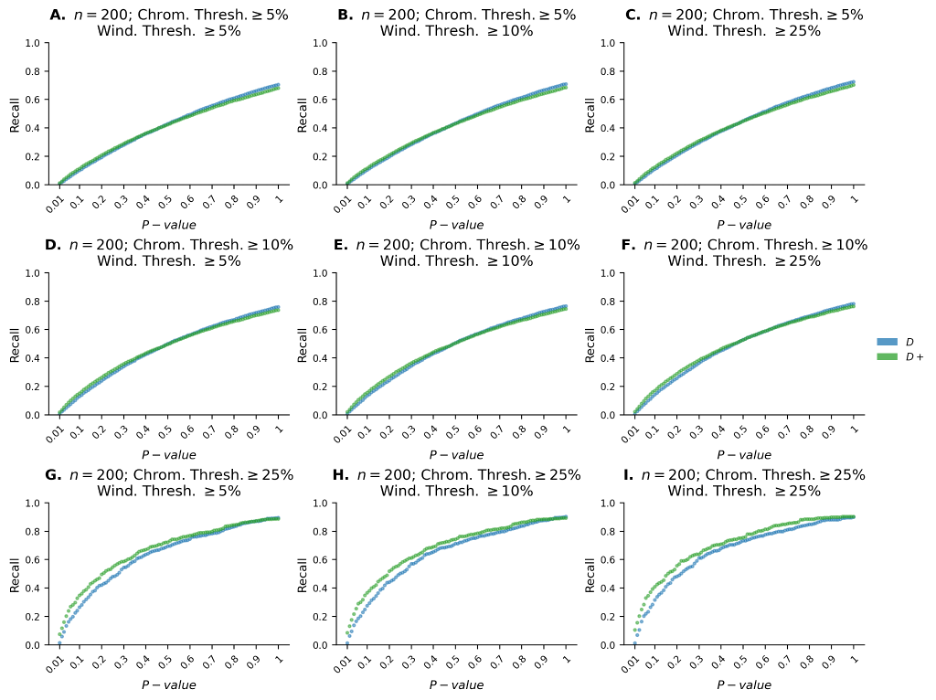

Supplement: S26 Fig — The Recall of D (blue) and D+ (green) were computed in non-overlapping 50 kb windows of 100 simulations of a 20 MB genome sampling 200 chromosomes from P1 and P2 and two chromosomes from P3 with an admixture proportion of 3% (f = 0.03). Using all pairwise combinations of requiring introgressed tracts to be present in at least 5% (top row), 10% (middle row), and 25% (bottom row) of sampled P2 chromosomes and requiring introgressed tracts to cover at least 5% (left column), 10% (middle column), and 25% (right column) of a 50kb window. Recall is shown as a function of the p-value (0.01–1) used to get a significant threshold value of D and D+. (TIFF) [file pgen.1010155.s026.tiff]
