# Supplementary material for: Leveraging shared ancestral variation to detect local introgression
Source: PLoS Genet. 2024 Jan 8;20(1):e1010155. doi: 10.1371/journal.pgen.1010155 (PMC10798638; doi:10.1371/journal.pgen.1010155)
Supplement: S1 Text — (DOCX) [file pgen.1010155.s028.docx]

**Supplementary Information**

**Methods**

**The modified Ragdale and Gravel (2019) model**

We also considered a more complex demographic history modified from the Ragsdale and Gravel (2019) model described in Peede et al 2022 (shown in S5 Fig). The simulations were done using msprime. The simulations include a mutation rate of 1.5*10^-8^ and a recombination rate of 10^-8^ base pairs per generation. The Ragsdale and Gravel (2019) model originally has continuous bidirectional migration. The modified model shown in S5 Fig has three discrete pulses of introgression from Neanderthals–one into Eurasians, and one into CEU and CHB respectively. These pulses are the midpoint of the two period of continuous bidirectional gene flow described in the original model.

**Calculating power for** $D$ **and** $D^{+}$ **genomewide**

We conducted 100 replicate simulations using msprime for the Instantaneous Unidirectional Admixture model and modified Ragsdale and Gravel (2019) model (shown in S5 Fig) of 100 Mb genomes using a sampling scheme of n=1 and n=100 monoploid genomes for the P_1_ and P_2_ populations and n=1 monoploid genome from P_3_ to represent realistic sampling of Neanderthals (P_3_). $D$ and $D^{+}$ were calculated using YRI as P_1_, Neanderthals as P_3_ and two separate analyses with CEU and then CHB as P_2_. This was done for admixture proportions of f = 1%, 2%, 3%, 4%, 5%, 6%, 7%, 8%, 9%, 10%, 20%, 30%, 40% and 50%. For each replicate simulation we built a bootstrapped distribution consisting of 1000 bootstrapped replicates. Each bootstrapped replicate was created from sampling with replacement 1000 windows of size 100 kb from the simulated genome and concatenating the windows to build a bootstrapped genome. The power of $D$ and $D^{+}$ was then determined by the proportion of replicates—out of 100 simulated replicates per admixture proportion—where the absolute value of $D$ or $D^{+}$ significantly differed from 0 by using the observed value from the replicate simulation and the bootstrap distribution consisting of 1000 bootstrapped replicates. Lastly, it should be noted that the power of $D$ and $D^{+}$ corresponds to the false positive rate of each statistic when there is no introgression.

**Adding sequencing error to simulated genomes**

First, to assess how many sequencing errors one would expect in a 50 kb region of the human genome we generated a synthetic genome consisting of 3 Gb and assumed the standard sequencing error rate used in of 0.001 as Durand et al. (2011), and randomly introduced errors independently in P_1_, P_2_, and P_1_ and P_2_—resulting in three sets of genomes with sequencing errors per replicate—and repeated such process 100 times. For each replicate we then calculated the number of sequencing errors present in a 50 kb region and plotted the distributions in S16 Fig. It is important to note that results in S16 Fig are representative of introducing sequencing errors to a hypothetical genome of 1X coverage. Additionally, even for a hypothetical genome of 1X coverage this is an overly conservative approach as all the sequencing errors would need to pass standard genomic quality control pipelines. S16 Fig highlights that for low coverage genomes sequencing error will impact any window-based approach for estimating site patterns. However, for higher coverage genomes with genomic quality control pipelines set in place to help avoid erroneous genotype calls we do not believe that sequencing error will have a large impact on window-based estimates of $D^{+}$. Next, to assess the effect of sequencing error on $D$ and $D^{+}$ we simulated 100 Mb genomes under the IUA model with no introgression. For the sample size n=1, we randomly selected 10,000 sites to introduce sequencing errors to. Here we assumed the same sequencing error rate of 1e-4 which showed that this rate of differential sequencing error between P_1_ and P_2_ is sufficient to produce false positives in $D$ on a genomic scale. Next, we randomly introduced sequencing errors independently in P_1_, P_2_, and P_1_ and P_2_, thus creating three sets of simulated genomes with sequencing errors for every original simulated genome. For each set of simulated genomes with sequencing errors we then calculated $D$ and $D^{+}$ in 50 kb windows and calculated the FPR as described in the “Calculating precision, recall and false positive rate in simulated human data” section in the Methods.

**Comparing performance of** $D$ **and** $D^{+}$ **for ILS and introgression**

We simulated 100,000 independent loci of size 10 kb, 20 kb, 30 kb, 40 kb, and 50 kb for admixture proportions of 0% and 3%. For each simulation we partitioned each locus by coalescent history, then only calculated $D$ and $D^{+}$ for the loci that had coalescent histories of ILS or introgression respectively. We plotted the distribution of $D$ and $D^{+}$ for each of the two coalescent histories (ILS or introgression) (S19 and S21 Figs). To test if the distributions follow a normal distribution, we also plotted the associated quantile-quantile (Q-Q) plots where we calculated the line of best fit between the theoretical quantiles and observed values using a least-squares regression and subsequently calculated the corresponding coefficient of determination (R^2^, S20 and S22 Figs).

**Comparing performance of** $D$ **and** $D^{+}$ **for different chromosome and window thresholds**

Here, we investigated the performance $D$ and $D^{+}$ by applying other window thresholds, i.e., requiring introgressed tracts to cover at least 5%, 10%, and 25% of a 50kb window, and chromosome thresholds, i.e., requiring introgressed tracts to be present in at least 5%, 10%, and 25% of sampled P_2_ chromosomes. We conducted 100 replicate simulations using the demographic model described in Fig 2 of the main text, for admixture proportion ($f$) of 0% and 3%, and a sequence length of 20 MB for two sampling schemes: (1) a single chromosome sampled from P_1_, P_2_ and P_3_ and (2) 200 chromosomes samples from P_1_ (representing 100 African individuals), 200 chromosomes sample from P_2_ (representing 100 Eurasian individuals), and 2 chromosomes sampled from P_3_ (representing 1 Neanderthal individual). To assess the performance of $D$ and $D^{+}$ we computed precision and recall in the same manner as outlined in the “Calculating precision, recall and false positive rate in simulated human data.” methods section in the main text. For sampling strategy (1), we assessed the performance of $D$ and $D^{+}$ for all of the aforementioned window thresholds (S24 Fig). For sampling strategy (2), we assessed the performance of $D$ and $D^{+}$ for all pairwise combinations of window and chromosome thresholds (S25-S26 Figs).


$D^{+}$ $D$ $D$ $D^{+}$ $D$ $D^{+}$ $D$ $D^{+}$ $f$ $D$ $D^{+}$ $D$ $D^{+}$ $f$ $D$ $D^{+}$ $D$ $D^{+}$ $D$ $D^{+}$ $D$ $D^{+}$ $f=2\%$ $f=5\%$ $f=10\%$ $D$ $D^{+}$ $D$ $D^{+}$ $D$ $D^{+}$ $D^{+}$ $D$ $d_{f}$ $D^{+}$ $D$ $d_{f}$ $D^{+}$ $f=10\%, 20\%, 30\%, 40\%$ $50\%$ $D^{+}$ $D^{+}$ $D^{+}$ $D^{+}$ $D$ $D^{+}$ $D$ $D^{+}$ $D$ $D^{+}$ $D^{+}$ $D_{ancestral}$ $D_{ancestral}$ $D$ $D^{+}$ $D$ $D^{+}$ $D$ $D$ $D$ $D+$ $D+$ $D+$ $D$ $D^{+}$ $D$ $D^{+}$ $f=0.03$ $D$ $D^{+}$ $D$ $D^{+}$ $D$ $D^{+}$ $f=0.03$ $D$ $D^{+}$ $D$ $D^{+}$ $D$ $D^{+}$ $f=0.03$ $D$ $D^{+}$ $D^{+}$ $D$ $N(ABBA)$ $N(BABA)$

$$D=\frac{N\left( ABBA \right)-N(BABA)}{N\left( ABBA \right)+N(BABA)}$$

$N(ABBA)$ $N(BABA)$ $D$ $N_{1}=N_{2}=N_{3}=N_{12}=N_{123}$

$$E\left[ T_{ABBA} \right]=f\left( T_{P3}-T_{GF} \right)+\left( 1-f \right)\left( 1-\frac{1}{2N} \right)^{T_{P3}-T_{P2}}\frac{2N}{3}+f\left( 1-\frac{1}{2N} \right)^{T_{P3}-T_{GF}}\frac{2N}{3}$$

$$E\left[ T_{BABA} \right]=\left( 1-f \right)\left( 1-\frac{1}{2N} \right)^{T_{P3}-T_{P2}}\frac{2N}{3}+f\left( 1-\frac{1}{2N} \right)^{T_{P3}-T_{GF}}\frac{2N}{3}$$

$D$

$$E\left[ D \right]=\frac{E\left[ T_{ABBA} \right]-E\left[ T_{BABA} \right]}{E\left[ T_{ABBA} \right]+E\left[ T_{BABA} \right]}$$

$$\left( 1-f \right)*\sum_{i=1}^{T_{P3}-T_{P2}} \left( Branch length at generation i \right)*P\left( Coalescence at generation i \right)$$

$$\left( 1-f \right)*\left( \sum_{i=1}^{T_{P3}-T_{P2}} \left( T_{P2}+i \right)*\frac{1}{2N}\left( 1-\frac{1}{2N} \right)^{i-1} \right)$$

$$\left( 1-f \right)*P\left( No coalescence of P_{1} and P_{2} before T_{P3} \right)*E\left[ Branch length in first coalescent event between lineages P_{1}, P_{2} and P_{3} \right]*P(P_{1} lineage coalesces in first coalescent event)$$

$$\left( 1-f \right)*\left( 1-\sum_{i=1}^{T_{P3}-T_{P2}} \frac{1}{2N}\left( 1-\frac{1}{2N} \right)^{i-1} \right)*\left( \frac{2N}{3}+T_{P3} \right)*\frac{2}{3}$$

$$\left( 1-f \right)*P\left( no coalescence of P_{1} and P_{2} before T_{P3} \right)*E\left[ Branch length in second coalescent event between lineages P_{1} and the ancestral lineage of P_{2} and P_{3} \right]*P(P_{1} lineage coalesces in second coalescent event)$$

$$\left( 1-f \right)*\left( 1-\sum_{i=1}^{T_{P3}-T_{P2}} \frac{1}{2N}\left( 1-\frac{1}{2N} \right)^{i-1} \right)*\left( 2N+\frac{2N}{3}+T_{P3} \right)*\frac{1}{3}$$

$$f*P\left( no coalescence for P_{2} and P_{3} before T_{GF} \right)*E\left[ branch length in first coalescent event between lineages P_{1}, P_{2} and P_{3} \right]*P(P_{1} lineage coalesces in first coalescent event)$$

$$f*\left( 1-\sum_{i=1}^{T_{P3}-T_{GF}} \frac{1}{2N}\left( 1-\frac{1}{2N} \right)^{i-1} \right)*\left( \frac{2N}{3}+T_{P3} \right)*\frac{2}{3}$$

$$f*P\left( no coalescence for P_{2} and P_{3} before T_{GF} \right)*E\left[ branch length in second coalescent event between lineages P1 and the ancestral lineage of P_{2} and P_{3} \right]*P(P_{1} lineage coalesces in second coalescent event)$$

$$f*\left( 1-\sum_{i=1}^{T_{P3}-T_{GF}} \frac{1}{2N}\left( 1-\frac{1}{2N} \right)^{i-1} \right)*\left( 2N+\frac{2N}{3}+T_{P3} \right)*\frac{1}{3}$$

$$f*P\left( coalescence for P_{2} and P_{3} before T_{GF} \right)*E\left[ branch length in coalescent event between lineages P_{1} and lineage (P_{2}, P_{3}) \right]$$

$$f*\left( \sum_{i=1}^{T_{P3}-T_{GF}} \frac{1}{2N}\left( 1-\frac{1}{2N} \right)^{i-1} \right)*(2N+T_{P3})$$

$$E[T_{BAAA}]=\left( 1-f \right)*\left( \left( \sum_{i=1}^{T_{P3}-T_{P2}} \left( T_{P2}+i \right)*\frac{1}{2N}\left( 1-\frac{1}{2N} \right)^{i-1} \right)+\left( \left( 1-\sum_{i=1}^{T_{P3}-T_{P2}} \frac{1}{2N}\left( 1-\frac{1}{2N} \right)^{i-1} \right)*\left( \frac{2N}{3}+T_{P3} \right)*\frac{2}{3} \right)+\left( \left( 1-\sum_{i=1}^{T_{P3}-T_{P2}} \frac{1}{2N}\left( 1-\frac{1}{2N} \right)^{i-1} \right)*\left( 2N+\frac{2N}{3}+T_{P3} \right)*\frac{1}{3} \right) \right)+f\left( \left( \left( 1-\sum_{i=1}^{T_{P3}-T_{GF}} \frac{1}{2N}\left( 1-\frac{1}{2N} \right)^{i-1} \right)*\left( \frac{2N}{3}+T_{P3} \right)*\frac{2}{3} \right)+\left( \left( 1-\sum_{i=1}^{T_{P3}-T_{GF}} \frac{1}{2N}\left( 1-\frac{1}{2N} \right)^{i-1} \right)*\left( 2N+\frac{2N}{3}+T_{P3} \right)*\frac{1}{3} \right)+\left( \left( \sum_{i=1}^{T_{P3}-T_{GF}} \frac{1}{2N}\left( 1-\frac{1}{2N} \right)^{i-1} \right)*\left( 2N+T_{P3} \right) \right) \right)$$

$$E[T_{BAAA}]=\left( 1-f \right)*\left( \left( \int_{i=0}^{T_{P3}-T_{P2}} (T_{P2}+i)\frac{1}{2N}e^{\frac{-i}{2N}}d_{i} \right)+\left( \left( e^{-\frac{TP3-TP2}{2N}} \right)*\left( \frac{2N}{3}+T_{P3} \right)*\frac{2}{3} \right)+\left( \left( e^{-\frac{TP3-TP2}{2N}} \right)*\left( 2N+\frac{2N}{3}+T_{P3} \right)*\frac{1}{3} \right) \right)+f\left( \left( \left( e^{-\frac{TP3-TGF}{2N}} \right)*\left( \frac{2N}{3}+T_{P3} \right)*\frac{2}{3} \right)+\left( \left( e^{-\frac{TP3-TGF}{2N}} \right)*\left( 2N+\frac{2N}{3}+T_{P3} \right)*\frac{1}{3} \right)+\left( \left( 1-e^{-\frac{TP3-TGF}{2N}} \right)*\left( 2N+T_{P3} \right) \right) \right)$$

$$E[T_{BAAA}]=\left( 1-f \right)*\left( \left( (-e^{\frac{-\left( TP3-TP2 \right)}{2N}}\left( 2N+\left( TP3-TP2 \right)+TP2 \right)+2N+TP2) \right)+\left( \left( e^{-\frac{TP3-TP2}{2N}} \right)*\left( \frac{2N}{3}+T_{P3} \right)*\frac{2}{3} \right)+\left( \left( e^{-\frac{TP3-TP2}{2N}} \right)*\left( 2N+\frac{2N}{3}+T_{P3} \right)*\frac{1}{3} \right) \right)+f\left( \left( \left( e^{-\frac{TP3-TGF}{2N}} \right)*\left( \frac{2N}{3}+T_{P3} \right)*\frac{2}{3} \right)+\left( \left( e^{-\frac{TP3-TGF}{2N}} \right)*\left( 2N+\frac{2N}{3}+T_{P3} \right)*\frac{1}{3} \right)+\left( \left( 1-e^{-\frac{TP3-TGF}{2N}} \right)*\left( 2N+T_{P3} \right) \right) \right)$$

$$E[T_{BAAA}]=\left( 1-f \right)*\left( \left( 2N+T_{P2} \right)+\left( \left( -e^{-\frac{T_{P3}-T_{P2}}{2N}} \right)*\left( \frac{2N}{3} \right) \right) \right)+f\left( \left( -e^{-\frac{T_{P3}-T_{GF}}{2N}} \right)*\left( \frac{2N}{3} \right)+2N+T_{P3} \right)$$

$$\left( 1-f \right)*\left( \left( \sum_{i=1}^{T_{P3}-T_{P2}} \left( T_{P2}+i \right)*\frac{1}{2N}\left( 1-\frac{1}{2N} \right)^{i-1} \right)+\left( \left( 1-\sum_{i=1}^{T_{P3}-T_{P2}} \frac{1}{2N}\left( 1-\frac{1}{2N} \right)^{i-1} \right)*\left( \frac{2N}{3}+T_{P3} \right)*\frac{2}{3} \right)+\left( \left( 1-\sum_{i=1}^{T_{P3}-T_{P2}} \frac{1}{2N}\left( 1-\frac{1}{2N} \right)^{i-1} \right)*\left( 2N+\frac{2N}{3}+T_{P3} \right)*\frac{1}{3} \right) \right)$$

$$f*P\left( no coalescence for P_{2} and P_{3} before T_{GF} \right)*E\left[ branch length in first coalescent event between lineages P_{1}, P_{2} and P_{3} \right]*P(P_{2} lineage coalesces in first coalescent event)$$

$$f*\left( 1-\sum_{i=1}^{T_{P3}-T_{GF}} \frac{1}{2N}\left( 1-\frac{1}{2N} \right)^{i-1} \right)*\left( \frac{2N}{3}+T_{P3} \right)*\frac{2}{3}$$

$$f*P\left( no coalescence for P_{2} and P_{3} before T_{GF} \right)*E\left[ branch length in second coalescent event between lineages P2 and the ancestral lineage of P_{1} and P_{3} \right]*P(P_{2} lineage coalesces in second coalescent event)$$

$$f*\left( 1-\sum_{i=1}^{T_{P3}-T_{GF}} \frac{1}{2N}\left( 1-\frac{1}{2N} \right)^{i-1} \right)*\left( 2N+\frac{2N}{3}+T_{P3} \right)*\frac{1}{3}$$

$$f*\left( \sum_{i=1}^{TP3-TGF} \left( TGF+i \right)*\frac{1}{2N}\left( 1-\frac{1}{2N} \right)^{i-1} \right)$$

$$E[T_{ABAA}]=\left( 1-f \right)*\left( \left( \sum_{i=1}^{T_{P3}-T_{P2}} \left( T_{P2}+i \right)*\frac{1}{2N}\left( 1-\frac{1}{2N} \right)^{i-1} \right)+\left( \left( 1-\sum_{i=1}^{T_{P3}-T_{P2}} \frac{1}{2N}\left( 1-\frac{1}{2N} \right)^{i-1} \right)*\left( \frac{2N}{3}+T_{P3} \right)*\frac{2}{3} \right)+\left( \left( 1-\sum_{i=1}^{T_{P3}-T_{P2}} \frac{1}{2N}\left( 1-\frac{1}{2N} \right)^{i-1} \right)*\left( 2N+\frac{2N}{3}+T_{P3} \right)*\frac{1}{3} \right) \right)+f\left( \left( \left( 1-\sum_{i=1}^{T_{P3}-T_{GF}} \frac{1}{2N}\left( 1-\frac{1}{2N} \right)^{i-1} \right)*\left( \frac{2N}{3}+T_{P3} \right)*\frac{2}{3} \right)+\left( \left( 1-\sum_{i=1}^{T_{P3}-T_{GF}} \frac{1}{2N}\left( 1-\frac{1}{2N} \right)^{i-1} \right)*\left( 2N+\frac{2N}{3}+T_{P3} \right)*\frac{1}{3} \right)+\left( \sum_{i=1}^{TP3-TGF} (TGF+i)*\frac{1}{2N}\left( 1-\frac{1}{2N} \right)^{i-1} \right) \right)$$

$$E[T_{ABAA}]=\left( 1-f \right)*\left( \left( \int_{i=0}^{T_{P3}-T_{P2}} (T_{P2}+i)\frac{1}{2N}e^{\frac{-i}{2N}}d_{i} \right)+\left( \left( e^{-\frac{TP3-TP2}{2N}} \right)*\left( \frac{2N}{3}+T_{P3} \right)*\frac{2}{3} \right)+\left( \left( e^{-\frac{TP3-TP2}{2N}} \right)*\left( 2N+\frac{2N}{3}+T_{P3} \right)*\frac{1}{3} \right) \right)+f\left( \left( \left( e^{-\frac{TP3-TGF}{2N}} \right)*\left( \frac{2N}{3}+T_{P3} \right)*\frac{2}{3} \right)+\left( \left( e^{-\frac{TP3-TGF}{2N}} \right)*\left( 2N+\frac{2N}{3}+T_{P3} \right)*\frac{1}{3} \right)+\left( \int_{i=0}^{T_{P3}-T_{GF}} (T_{GF}+i)\frac{1}{2N}e^{\frac{-i}{2N}}d_{i} \right) \right)$$

$$E[T_{ABAA}]=\left( 1-f \right)*\left( \left( (-e^{\frac{-\left( TP3-TP2 \right)}{2N}}\left( 2N+\left( TP3-TP2 \right)+TP2 \right)+2N+TP2) \right)+\left( \left( e^{-\frac{TP3-TP2}{2N}} \right)*\left( \frac{2N}{3}+T_{P3} \right)*\frac{2}{3} \right)+\left( \left( e^{-\frac{TP3-TP2}{2N}} \right)*\left( 2N+\frac{2N}{3}+T_{P3} \right)*\frac{1}{3} \right) \right)+f\left( \left( \left( e^{-\frac{TP3-TGF}{2N}} \right)*\left( \frac{2N}{3}+T_{P3} \right)*\frac{2}{3} \right)+\left( \left( e^{-\frac{TP3-TGF}{2N}} \right)*\left( 2N+\frac{2N}{3}+T_{P3} \right)*\frac{1}{3} \right)+\left( (-e^{\frac{-\left( TP3-TGF \right)}{2N}}\left( 2N+\left( TP3-TGF \right)+TGF \right)+2N+TGF) \right) \right)$$

$$E\left[ T_{ABAA} \right]=\left( 1-f \right)*\left( \left( 2N+T_{P2} \right)+\left( \left( -e^{-\frac{T_{P3}-T_{P2}}{2N}} \right)*\left( \frac{2N}{3} \right) \right) \right)+f\left( -\left( e^{-\frac{T_{P3}-T_{GF}}{2N}} \right)\frac{2N}{3}+2N+T_{GF} \right)$$
