# Supplementary material for: Leveraging shared ancestral variation to detect local introgression
Source: PLoS Genet. 2024 Jan 8;20(1):e1010155. doi: 10.1371/journal.pgen.1010155 (PMC10798638; doi:10.1371/journal.pgen.1010155)
Supplement: S1 Appendix — (DOCX) [file pgen.1010155.s029.docx]

**S1 Appendix. Derivation of** $D^{+}$**.**

Under the Instantaneous Unidirectional Admixture (IUA) model, (4) and [[5]](https://paperpile.com/c/qmOSMa/elzX7) propose the $D$ statistic is to infer patterns of gene flow. It quantifies differences in the number of site patterns $N(ABBA)$ and $N(BABA)$:

$$D=\frac{N\left( ABBA \right)-N(BABA)}{N\left( ABBA \right)+N(BABA)}$$

Where $N(ABBA)$ and $N(BABA)$ are the number of sites that have an ABBA or a BABA pattern. In an ABBA pattern, the lineages P_2_ and P_3_ share a derived site. Under the BABA pattern, the lineages P_1_ and P_3_ share a derived site.

To estimate $D$, [[5]](https://paperpile.com/c/qmOSMa/elzX7) assumed that the effective population sizes are equal across the whole demographic scenario. Therefore $N_{1}=N_{2}=N_{3}=N_{12}=N_{123}$. [[5]](https://paperpile.com/c/qmOSMa/elzX7) derived the probability of obtaining an ABBA or a BABA site, where the probability of obtaining those sites is equal to the product of the mutation rate times the expected length of the branch where a mutation would produce an ABBA or BABA site, respectively. Based on [[5]](https://paperpile.com/c/qmOSMa/elzX7), the expected length of the branch *T_ABBA_* where a mutation would produce an ABBA site is equal to:

$$E\left[ T_{ABBA} \right]=f\left( T_{P3}-T_{GF} \right)+\left( 1-f \right)\left( 1-\frac{1}{2N} \right)^{T_{P3}-T_{P2}}\frac{2N}{3}+f\left( 1-\frac{1}{2N} \right)^{T_{P3}-T_{GF}}\frac{2N}{3}$$

And:

$$E\left[ T_{BABA} \right]=\left( 1-f \right)\left( 1-\frac{1}{2N} \right)^{T_{P3}-T_{P2}}\frac{2N}{3}+f\left( 1-\frac{1}{2N} \right)^{T_{P3}-T_{GF}}\frac{2N}{3}$$

Using those expected branch lengths, the expected value of the $D$ statistic can be calculated as:

$$E\left[ D \right]=\frac{E\left[ T_{ABBA} \right]-E\left[ T_{BABA} \right]}{E\left[ T_{ABBA} \right]+E\left[ T_{BABA} \right]}$$

Now we will derive the expected lengths of the branches where a mutation would create a BAAA or an ABAA site. A BAAA site is one where there is a derived allele in the P_1_ individual and an ABAA site only contains a derived allele in the P_2_ individual.

***BAAA sites***

In this section we show how to estimate the expected lengths of branches that produce a BAAA site under the IUA model. The expected branch lengths are equal to the sum of the contributions from six different scenarios that could lead to the coalescence of the lineage P_1_:


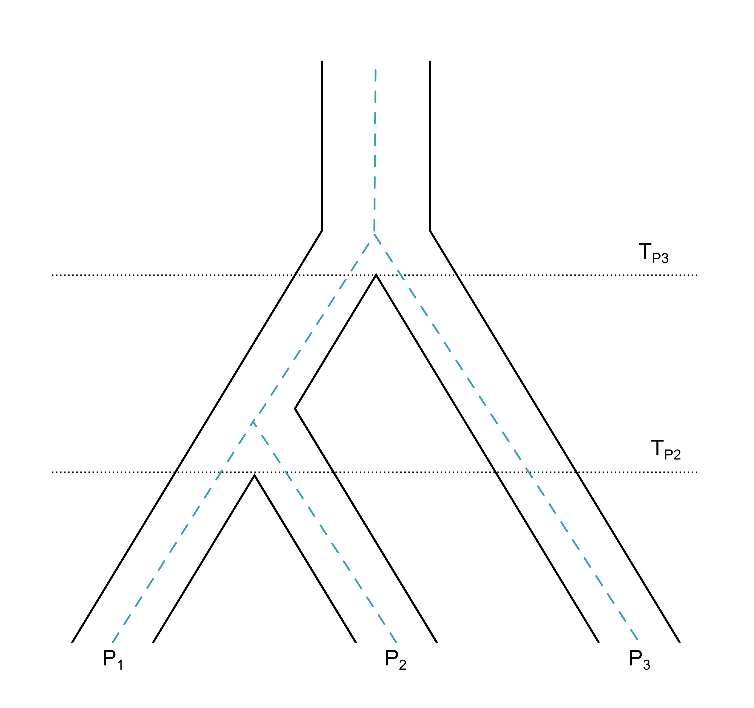


**A1 Fig**. Scenario 1 for a BAAA site.

1) There was no gene flow from P_3_ to P_2_. The P_1_ lineage coalesces with the P_2_ lineage between times T_P3_ and T_P2_:

$$\left( 1-f \right)*\sum_{i=1}^{T_{P3}-T_{P2}} \left( Branch length at generation i \right)*P\left( Coalescence at generation i \right)$$

$$\left( 1-f \right)*\left( \sum_{i=1}^{T_{P3}-T_{P2}} \left( T_{P2}+i \right)*\frac{1}{2N}\left( 1-\frac{1}{2N} \right)^{i-1} \right)$$

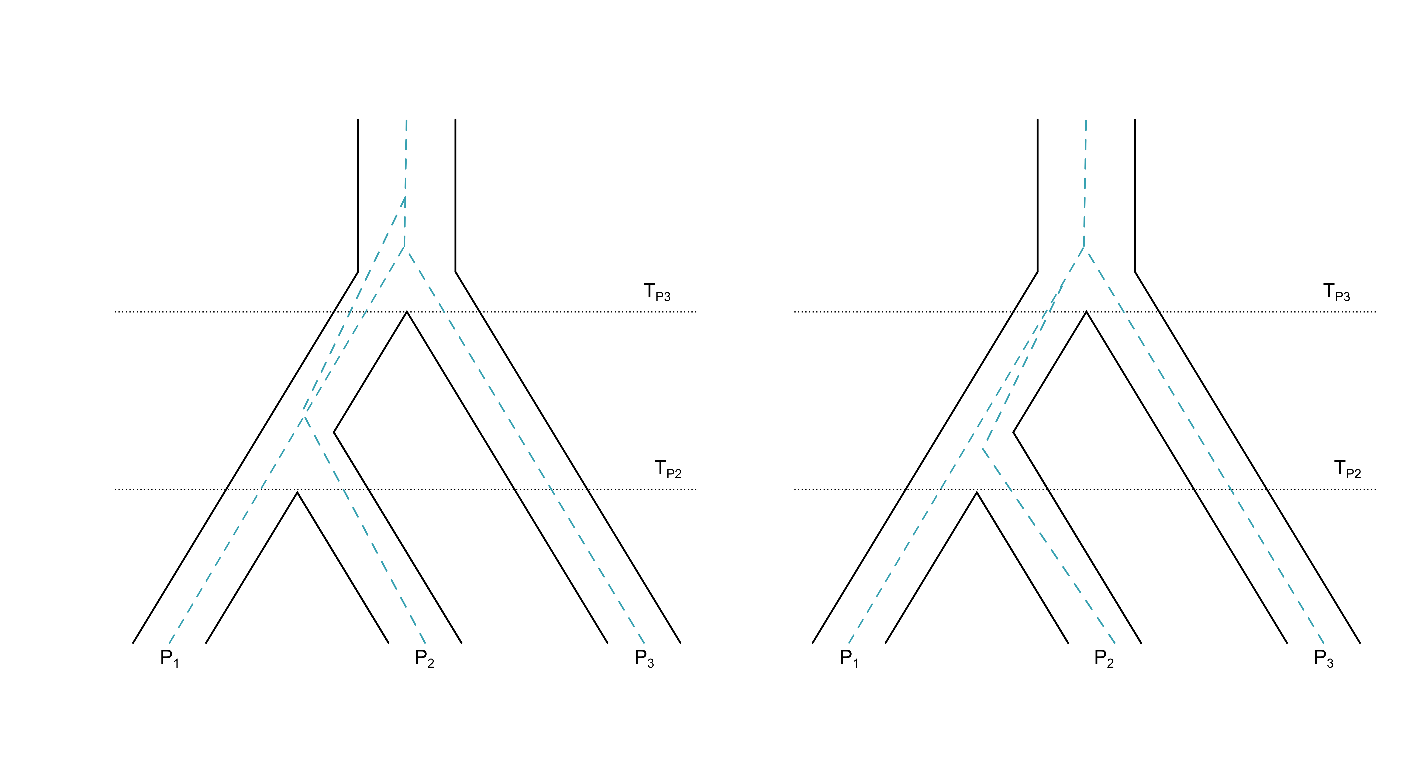


**A2 Fig.** Scenario 2 for a BAAA site.

2) There was no gene flow from P_3_ to P_2_. The P_1_ lineage coalesces with either P_2_ or P_3_ in the first coalescent event that takes place after T_P3_ going backwards into the past.

$$\left( 1-f \right)*P\left( No coalescence of P_{1} and P_{2} before T_{P3} \right)*E\left[ Branch length in first coalescent event between lineages P_{1}, P_{2} and P_{3} \right]*P(P_{1} lineage coalesces in first coalescent event)$$

$$\left( 1-f \right)*\left( 1-\sum_{i=1}^{T_{P3}-T_{P2}} \frac{1}{2N}\left( 1-\frac{1}{2N} \right)^{i-1} \right)*\left( \frac{2N}{3}+T_{P3} \right)*\frac{2}{3}$$

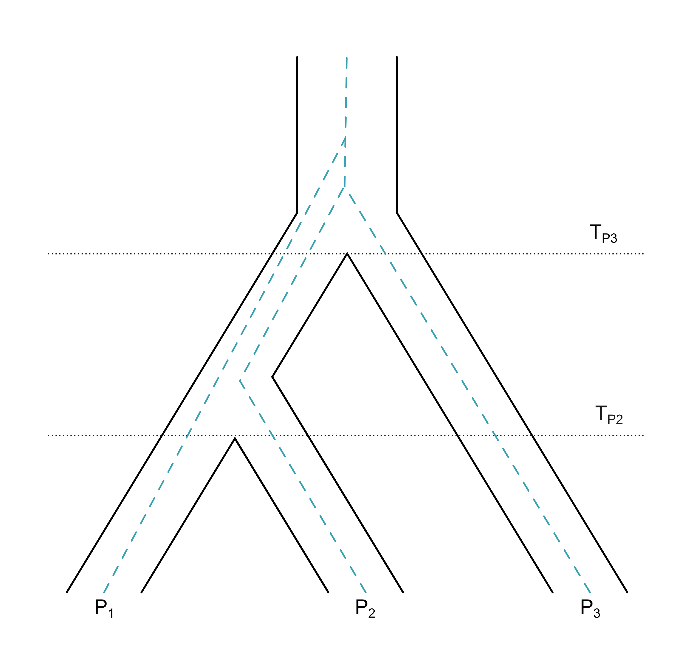


**A3 Fig**. Scenario 3 for a BAAA site.

3) There was no gene flow from P_3_ to P_2_. The P_1_ lineage coalesces with the ancestral lineage of P_2_ and P_3_ in the second coalescent event that takes place after T_P3_ going backwards into the past.

$$\left( 1-f \right)*P\left( no coalescence of P_{1} and P_{2} before T_{P3} \right)*E\left[ \begin{aligned} Branch length in second coalescent event between \\ lineages P_{1} and the ancestral lineage of P_{2} and P_{3} \end{aligned} \right]*P(P_{1} lineage coalesces in second coalescent event)$$

$$\left( 1-f \right)*\left( 1-\sum_{i=1}^{T_{P3}-T_{P2}} \frac{1}{2N}\left( 1-\frac{1}{2N} \right)^{i-1} \right)*\left( 2N+\frac{2N}{3}+T_{P3} \right)*\frac{1}{3}$$

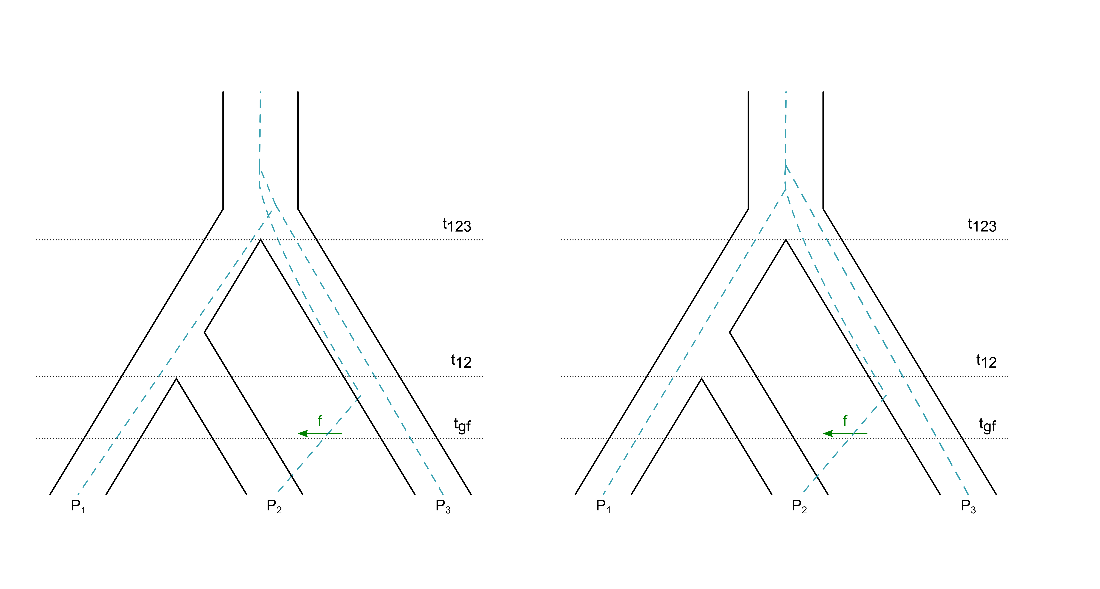


**A4 Fig**. Scenario 4 for a BAAA site.

4) There was gene flow from P_3_ to P_2_. The P_3_ and P_2_ lineages did not coalesce between times T_P3_ and T_GF_. The lineage P_1_ coalesces in the first coalescent event after T_P3_ going backwards into the past.

$$f*P\left( no coalescence for P_{2} and P_{3} before T_{GF} \right)*E\left[ branch length in first coalescent event between lineages P_{1}, P_{2} and P_{3} \right]*P(P_{1} lineage coalesces in first coalescent event)$$

$$f*\left( 1-\sum_{i=1}^{T_{P3}-T_{GF}} \frac{1}{2N}\left( 1-\frac{1}{2N} \right)^{i-1} \right)*\left( \frac{2N}{3}+T_{P3} \right)*\frac{2}{3}$$

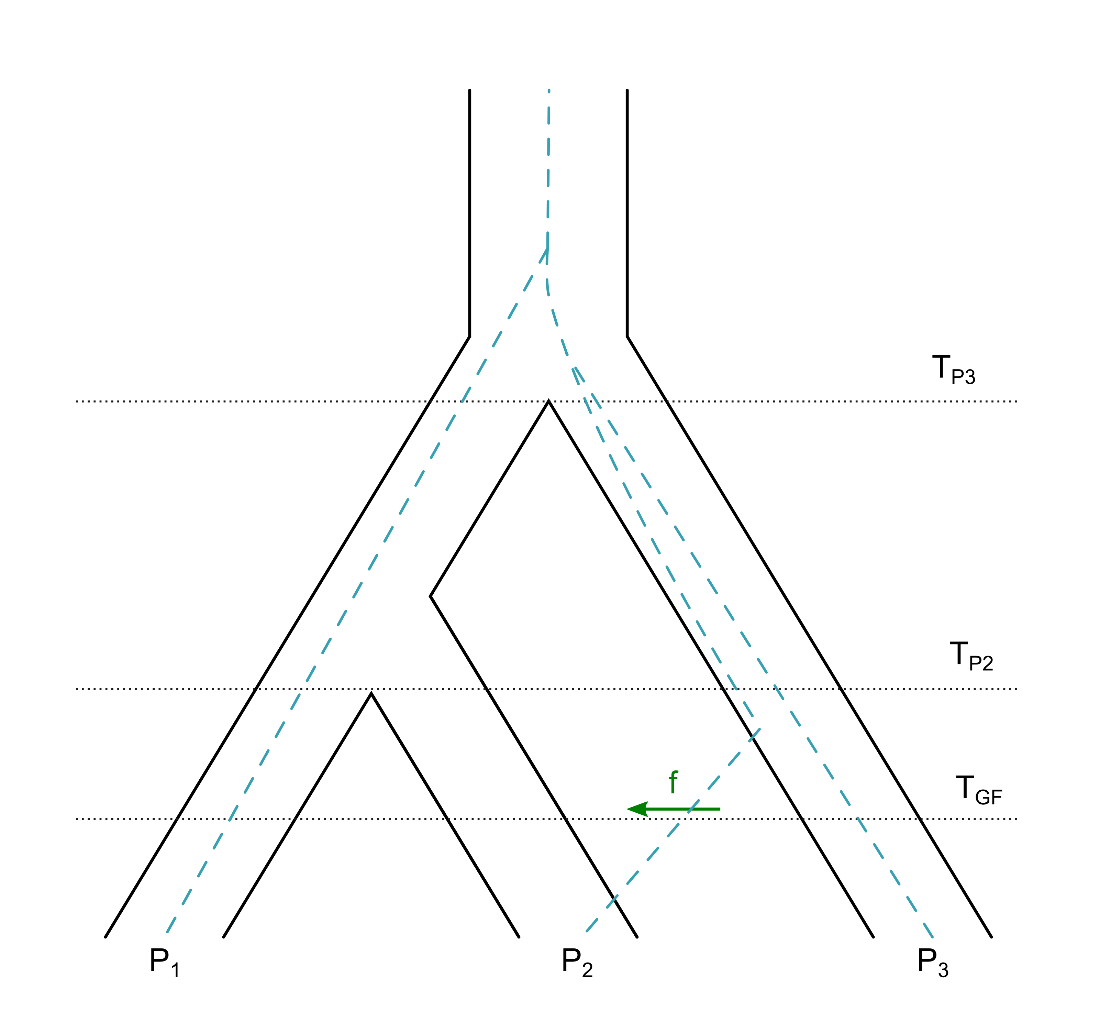


**A5 Fig**. Scenario 5 for a BAAA site.

5) There was gene flow from P_3_ to P_2_. The P_3_ and P_2_ lineages did not coalesce between times T_P3_ and T_GF_. The P_1_ lineage coalesces with the ancestral lineage of P_2_ and P_3_ in the second coalescent event that takes place after T_P3_ going backwards into the past.

$$f*P\left( no coalescence for P_{2} and P_{3} before T_{GF} \right)*E\left[ \begin{aligned} branch length in second coalescent event between \\ lineages P1 and theancestral lineage of P_{2} and P_{3} \end{aligned} \right]*P(P_{1} lineage coalesces in second coalescent event)$$

$$f*\left( 1-\sum_{i=1}^{T_{P3}-T_{GF}} \frac{1}{2N}\left( 1-\frac{1}{2N} \right)^{i-1} \right)*\left( 2N+\frac{2N}{3}+T_{P3} \right)*\frac{1}{3}$$

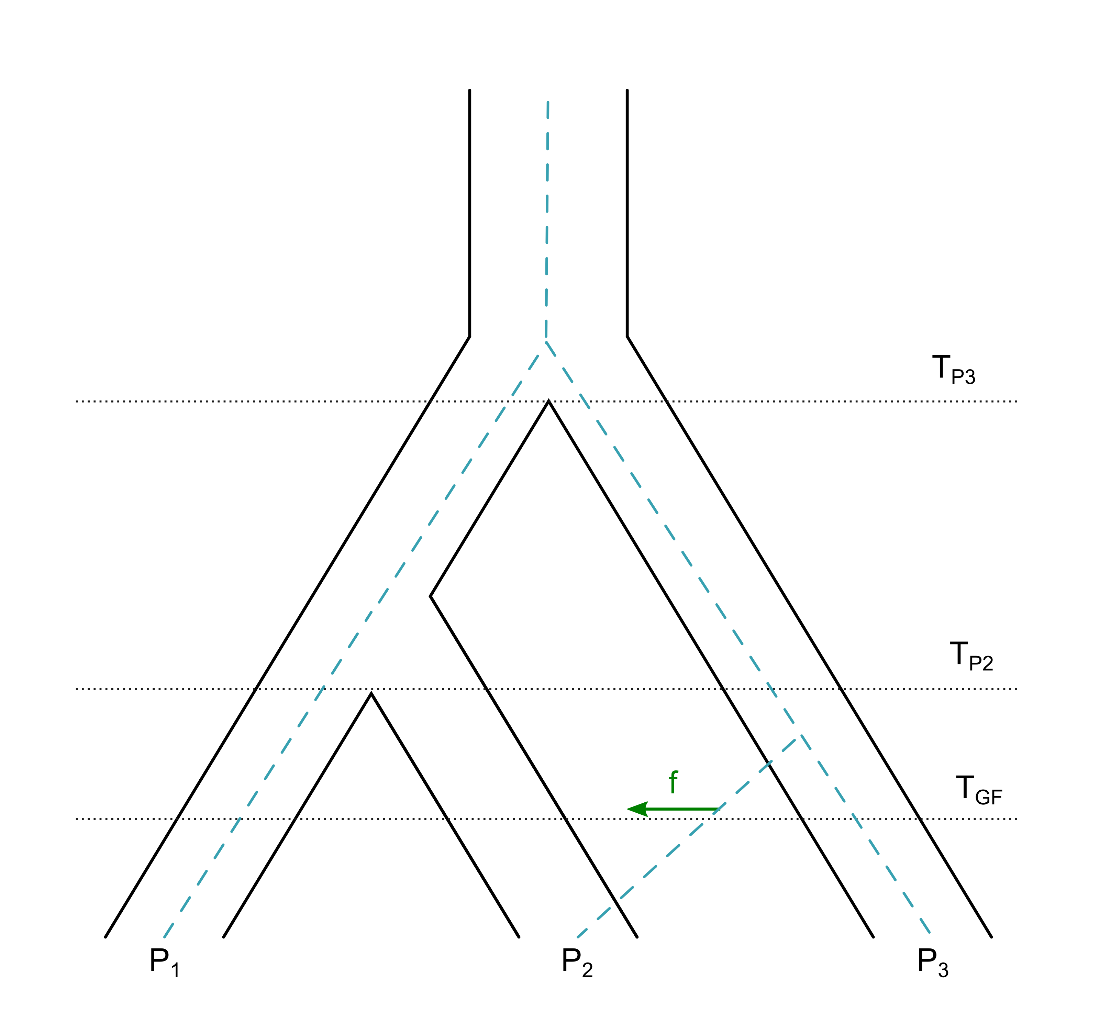


**A6 Fig**. Scenario 6 for a BAAA site.

6) There was gene flow from P_3_ to P_2_. P_2_ and P_3_ coalesce between T_P3_ and T_GF_. The lineage P_1_ coalesces with the lineage ancestral to P_2_ and P_3_ after T_P3_ going backwards into the past.

$$f*P\left( coalescence for P_{2} and P_{3} before T_{GF} \right)*E\left[ branch length in coalescent event between lineages P_{1} and lineage (P_{2}, P_{3}) \right]$$

$$f*\left( \sum_{i=1}^{T_{P3}-T_{GF}} \frac{1}{2N}\left( 1-\frac{1}{2N} \right)^{i-1} \right)*(2N+T_{P3})$$

If we sum those six contributions, we get:

$$E[T_{BAAA}]=\left( 1-f \right)*\left( \left( \sum_{i=1}^{T_{P3}-T_{P2}} \left( T_{P2}+i \right)*\frac{1}{2N}\left( 1-\frac{1}{2N} \right)^{i-1} \right)+\left( \left( 1-\sum_{i=1}^{T_{P3}-T_{P2}} \frac{1}{2N}\left( 1-\frac{1}{2N} \right)^{i-1} \right)*\left( \frac{2N}{3}+T_{P3} \right)*\frac{2}{3} \right)+\left( \left( 1-\sum_{i=1}^{T_{P3}-T_{P2}} \frac{1}{2N}\left( 1-\frac{1}{2N} \right)^{i-1} \right)*\left( 2N+\frac{2N}{3}+T_{P3} \right)*\frac{1}{3} \right) \right)+f\left( \left( \left( 1-\sum_{i=1}^{T_{P3}-T_{GF}} \frac{1}{2N}\left( 1-\frac{1}{2N} \right)^{i-1} \right)*\left( \frac{2N}{3}+T_{P3} \right)*\frac{2}{3} \right)+\left( \left( 1-\sum_{i=1}^{T_{P3}-T_{GF}} \frac{1}{2N}\left( 1-\frac{1}{2N} \right)^{i-1} \right)*\left( 2N+\frac{2N}{3}+T_{P3} \right)*\frac{1}{3} \right)+\left( \left( \sum_{i=1}^{T_{P3}-T_{GF}} \frac{1}{2N}\left( 1-\frac{1}{2N} \right)^{i-1} \right)*\left( 2N+T_{P3} \right) \right) \right)$$

We can replace some of the terms in that equation using an exponential function. This simplifies the past equation to:

$$E[T_{BAAA}]=\left( 1-f \right)*\left( \left( \int_{i=0}^{T_{P3}-T_{P2}} (T_{P2}+i)\frac{1}{2N}e^{\frac{-i}{2N}}d_{i} \right)+\left( \left( e^{-\frac{TP3-TP2}{2N}} \right)*\left( \frac{2N}{3}+T_{P3} \right)*\frac{2}{3} \right)+\left( \left( e^{-\frac{TP3-TP2}{2N}} \right)*\left( 2N+\frac{2N}{3}+T_{P3} \right)*\frac{1}{3} \right) \right)+f\left( \left( \left( e^{-\frac{TP3-TGF}{2N}} \right)*\left( \frac{2N}{3}+T_{P3} \right)*\frac{2}{3} \right)+\left( \left( e^{-\frac{TP3-TGF}{2N}} \right)*\left( 2N+\frac{2N}{3}+T_{P3} \right)*\frac{1}{3} \right)+\left( \left( 1-e^{-\frac{TP3-TGF}{2N}} \right)*\left( 2N+T_{P3} \right) \right) \right)$$

And solving the integral from the first term, we get:

$$E[T_{BAAA}]=\left( 1-f \right)*\left( \left( (-e^{\frac{-\left( T_{P3}-T_{P2} \right)}{2N}}\left( 2N+\left( T_{P3}-T_{P2} \right)+T_{P2} \right)+2N+T_{P2}) \right)+\left( \left( e^{-\frac{T_{P3}-T_{P2}}{2N}} \right)*\left( \frac{2N}{3}+T_{P3} \right)*\frac{2}{3} \right)+\left( \left( e^{-\frac{T_{P3}-T_{P2}}{2N}} \right)*\left( 2N+\frac{2N}{3}+T_{P3} \right)*\frac{1}{3} \right) \right)+f\left( \left( \left( e^{-\frac{T_{P3}-T_{GF}}{2N}} \right)*\left( \frac{2N}{3}+T_{P3} \right)*\frac{2}{3} \right)+\left( \left( e^{-\frac{T_{P3}-T_{GF}}{2N}} \right)*\left( 2N+\frac{2N}{3}+T_{P3} \right)*\frac{1}{3} \right)+\left( \left( 1-e^{-\frac{T_{P3}-T_{GF}}{2N}} \right)*\left( 2N+T_{P3} \right) \right) \right)$$

We can simplify this equation to get:

$$E[T_{BAAA}]=\left( 1-f \right)*\left( \left( 2N+T_{P2} \right)+\left( \left( -e^{-\frac{T_{P3}-T_{P2}}{2N}} \right)*\left( \frac{2N}{3} \right) \right) \right)+f\left( \left( -e^{-\frac{T_{P3}-T_{GF}}{2N}} \right)*\left( \frac{2N}{3} \right)+2N+T_{P3} \right)$$

***ABAA sites***

To calculate the branch lengths of the ABAA sites, we need to also calculate the contributions from six different scenarios. The calculations of the three scenarios without gene flow are equal to those of the BAAA sites:

$$\left( 1-f \right)*\left( \left( \sum_{i=1}^{T_{P3}-T_{P2}} \left( T_{P2}+i \right)*\frac{1}{2N}\left( 1-\frac{1}{2N} \right)^{i-1} \right)+\left( \left( 1-\sum_{i=1}^{T_{P3}-T_{P2}} \frac{1}{2N}\left( 1-\frac{1}{2N} \right)^{i-1} \right)*\left( \frac{2N}{3}+T_{P3} \right)*\frac{2}{3} \right)+\left( \left( 1-\sum_{i=1}^{T_{P3}-T_{P2}} \frac{1}{2N}\left( 1-\frac{1}{2N} \right)^{i-1} \right)*\left( 2N+\frac{2N}{3}+T_{P3} \right)*\frac{1}{3} \right) \right)$$

The contributions of the three scenarios with gene flow are:

1) There was gene flow from P_3_ to P_2_. The P_3_ and P_2_ lineages did not coalesce between times T_P3_ and T_GF_. The lineage P_2_ coalesces in the first coalescent event after T_P3_ going backwards into the past.

$$f*P\left( no coalescence for P_{2} and P_{3} before T_{GF} \right)*E\left[ branch length in first coalescent event between lineages P_{1}, P_{2} and P_{3} \right]*P(P_{2} lineage coalesces in first coalescent event)$$

$$f*\left( 1-\sum_{i=1}^{T_{P3}-T_{GF}} \frac{1}{2N}\left( 1-\frac{1}{2N} \right)^{i-1} \right)*\left( \frac{2N}{3}+T_{P3} \right)*\frac{2}{3}$$

2) There was gene flow from P_3_ to P_2_. The P_3_ and P_2_ lineages did not coalesce between times T_P3_ and T_GF_. The P_2_ lineage coalesces with the ancestral lineage of P_1_ and P_3_ in the second coalescent event that takes place after T_P3_ going backwards into the past.

$$f*P\left( no coalescence for P_{2} and P_{3} before T_{GF} \right)*E\left[ \begin{aligned} branch length in second coalescent event between \\ lineages P2 and the ancestral lineage of P_{1} and P_{3} \end{aligned} \right]*P(P_{2} lineage coalesces in second coalescent event)$$

$$f*\left( 1-\sum_{i=1}^{T_{P3}-T_{GF}} \frac{1}{2N}\left( 1-\frac{1}{2N} \right)^{i-1} \right)*\left( 2N+\frac{2N}{3}+T_{P3} \right)*\frac{1}{3}$$

3) There was gene flow from P3 to P2. The lineages P2 and P3 coalesce between T_GF_ and T_P3_.

$$f*\left( \sum_{i=1}^{T_{P3}-T_{GF}} \left( T_{GF}+i \right)*\frac{1}{2N}\left( 1-\frac{1}{2N} \right)^{i-1} \right)$$

Therefore, when we put it all together, we get:

$$E[T_{ABAA}]=\left( 1-f \right)*\left( \left( \sum_{i=1}^{T_{P3}-T_{P2}} \left( T_{P2}+i \right)*\frac{1}{2N}\left( 1-\frac{1}{2N} \right)^{i-1} \right)+\left( \left( 1-\sum_{i=1}^{T_{P3}-T_{P2}} \frac{1}{2N}\left( 1-\frac{1}{2N} \right)^{i-1} \right)*\left( \frac{2N}{3}+T_{P3} \right)*\frac{2}{3} \right)+\left( \left( 1-\sum_{i=1}^{T_{P3}-T_{P2}} \frac{1}{2N}\left( 1-\frac{1}{2N} \right)^{i-1} \right)*\left( 2N+\frac{2N}{3}+T_{P3} \right)*\frac{1}{3} \right) \right)+f\left( \left( \left( 1-\sum_{i=1}^{T_{P3}-T_{GF}} \frac{1}{2N}\left( 1-\frac{1}{2N} \right)^{i-1} \right)*\left( \frac{2N}{3}+T_{P3} \right)*\frac{2}{3} \right)+\left( \left( 1-\sum_{i=1}^{T_{P3}-T_{GF}} \frac{1}{2N}\left( 1-\frac{1}{2N} \right)^{i-1} \right)*\left( 2N+\frac{2N}{3}+T_{P3} \right)*\frac{1}{3} \right)+\left( \sum_{i=1}^{T_{P3}-T_{GF}} \left( T_{GF}+i \right)*\frac{1}{2N}\left( 1-\frac{1}{2N} \right)^{i-1} \right) \right)$$

Replacing some of the terms in that equation using an exponential function, we obtain:

$$E[T_{ABAA}]=\left( 1-f \right)*\left( \left( \int_{i=0}^{T_{P3}-T_{P2}} (T_{P2}+i)\frac{1}{2N}e^{\frac{-i}{2N}}d_{i} \right)+\left( \left( e^{-T_{P3}-T_{P2}} \right)*\left( \frac{2N}{3}+T_{P3} \right)*\frac{2}{3} \right)+\left( \left( e^{-\frac{T_{P3}-T_{P2}}{2N}} \right)*\left( 2N+\frac{2N}{3}+T_{P3} \right)*\frac{1}{3} \right) \right)+f\left( \left( \left( e^{-\frac{T_{P3}-T_{GF}}{2N}} \right)*\left( \frac{2N}{3}+T_{P3} \right)*\frac{2}{3} \right)+\left( \left( e^{-\frac{T_{P3}-T_{GF}}{2N}} \right)*\left( 2N+\frac{2N}{3}+T_{P3} \right)*\frac{1}{3} \right)+\left( \int_{i=0}^{T_{P3}-T_{GF}} (T_{GF}+i)\frac{1}{2N}e^{\frac{-i}{2N}}d_{i} \right) \right)$$

After solving the integrals, we get:

$$E[T_{ABAA}]=\left( 1-f \right)*\left( \left( (-e^{\frac{-\left( T_{P3}-T_{P2} \right)}{2N}}\left( 2N+\left( T_{P3}-T_{P2} \right)+T_{P2} \right)+2N+T_{P2}) \right)+\left( \left( e^{-\frac{T_{P3}-T_{P2}}{2N}} \right)*\left( \frac{2N}{3}+T_{P3} \right)*\frac{2}{3} \right)+\left( \left( e^{-\frac{T_{P3}-T_{P2}}{2N}} \right)*\left( 2N+\frac{2N}{3}+T_{P3} \right)*\frac{1}{3} \right) \right)+f\left( \left( \left( e^{-\frac{T_{P3}-T_{GF}}{2N}} \right)*\left( \frac{2N}{3}+T_{P3} \right)*\frac{2}{3} \right)+\left( \left( e^{-\frac{T_{P3}-T_{GF}}{2N}} \right)*\left( 2N+\frac{2N}{3}+T_{P3} \right)*\frac{1}{3} \right)+\left( (-e^{\frac{-\left( T_{P3}-T_{GF} \right)}{2N}}\left( 2N+\left( T_{P3}-T_{GF} \right)+T_{GF} \right)+2N+T_{GF}) \right) \right)$$

If we simplify this equation, we get:

$$E\left[ T_{ABAA} \right]=\left( 1-f \right)*\left( \left( 2N+T_{P2} \right)+\left( \left( -e^{-\frac{T_{P3}-T_{P2}}{2N}} \right)*\left( \frac{2N}{3} \right) \right) \right)+f\left( -\left( e^{-\frac{T_{P3}-T_{GF}}{2N}} \right)\frac{2N}{3}+2N+T_{GF} \right)$$
